# Genome-wide analysis of the *Hsp*20 gene family in soybean: comprehensive sequence, genomic organization and expression profile analysis under abiotic and biotic stresses

**DOI:** 10.1186/1471-2164-14-577

**Published:** 2013-08-28

**Authors:** Valéria S Lopes-Caitar, Mayra CCG de Carvalho, Luana M Darben, Marcia K Kuwahara, Alexandre L Nepomuceno, Waldir P Dias, Ricardo V Abdelnoor, Francismar C Marcelino-Guimarães

**Affiliations:** 1 Department of Biochemistry and Biotechnology, Londrina State University, Londrina, Brazil; 2 Northern Parana State University, Bandeirantes, Brazil; 3 Maringa State University, Maringa, Brazil; 4 Brazilian Agricultural Research Corporation’s – EMBRAPA Soybean, Londrina, Brazil

**Keywords:** Soybean, Small heat shock proteins, *Meloidogyne javanica*, *Cis*-elements

## Abstract

**Background:**

The *Hsp*20 genes are associated with stress caused by HS and other abiotic factors, but have recently been found to be associated with the response to biotic stresses. These genes represent the most abundant class among the HSPs in plants, but little is known about this gene family in soybean. Because of their apparent multifunctionality, these proteins are promising targets for developing crop varieties that are better adapted to biotic and abiotic stresses. Thus, in the present study an *in silico* identification of Gm*Hsp*20 gene family members was performed, and the genes were characterized and subjected to *in vivo* expression analysis under biotic and abiotic stresses.

**Results:**

A search of the available soybean genome databases revealed 51 gene models as potential Gm*Hsp*20 candidates. The 51 Gm*Hsp*20 genes were distributed across a total of 15 subfamilies where a specific predicted secondary structure was identified. Based on *in vivo* analysis, only 47 soybean *Hsp*20 genes were responsive to heat shock stress. Among the Gm*Hsp*20 genes that were potentials HSR, five were also cold-induced, and another five, in addition to one Gm*Acd* gene, were responsive to *Meloidogyne javanica* infection. Furthermore, one predicted Gm*Hsp*20 was shown to be responsive only to nematode infection; no expression change was detected under other stress conditions. Some of the biotic stress-responsive Gm*Hsp*20 genes exhibited a divergent expression pattern between resistant and susceptible soybean genotypes under *M. javanica* infection. The putative regulatory elements presenting some conservation level in the Gm*Hsp*20 promoters included HSE, W-box, CAAT box, and TA-rich elements. Some of these putative elements showed a unique occurrence pattern among genes responsive to nematode infection.

**Conclusions:**

The evolution of *Hsp*20 family in soybean genome has most likely involved a total of 23 gene duplications. The obtained expression profiles revealed that the majority of the 51 Gm*Hsp*20 candidates are induced under HT, but other members of this family could also be involved in normal cellular functions, unrelated to HT. Some of the Gm*Hsp*20 genes might be specialized to respond to nematode stress, and the predicted promoter structure of these genes seems to have a particular conserved pattern related to their biological function.

## Background

Plants inevitably interact with climatic factors and are often subjected to different types of biotic and abiotic stresses. Environmental stress conditions, such as those related to drought, flooding, salinity, cold, heat, chemical substances derived from human activities and pathogens, have adverse effects on plant growth and crop yields [1,2].

Temperature is one type of stress that greatly affects crop production around the world; however, additional stress factors may also act either separately or simultaneously and ultimately place the plant under combined stresses, causing cell damage and the production of secondary stresses, such as osmotic or oxidative stress [1,2]. As part of a biological system, plants are also attacked by different pests and pathogens. The diseases caused by root nematode parasites belonging to different genera, such as *Meloidogyne* spp., and the fungus *Phakopsora pachyrhizi*, which causes Asian Soybean Rust disease [3], have been contributing to decreases in soybean yields, especially in tropical and subtropical regions.

Plants are sessile organisms that are not able to avoid exposure to adverse effects. However, they can supplant such exposure through the evolution of different morphological, molecular and physiological mechanisms or adaptations [4]. Heat shock proteins are often associated with plant responses to cold stress, heavy metals and reactive oxygen species (ROS) [5]. Heat shock proteins (HSPs) have also recently been found to be associated with the plant response to infection by pathogens such as nematodes [6-9], bacteria [10,11] and fungi [12,13]. The signals or specific factors that trigger the expression of *Hsp*s genes during biotic stress are currently unknown, but the metabolic changes resulting from pathogen attack can generate similar signals or stimuli as those observed under abiotic stress activation [14,15].

The HSPs were first identified in *Drosophila melanogaster* in the response to heat shock stress [16]. These proteins are grouped into high molecular weight protein families, comprising the HSP100, HSP90, HSP70/DnaK and HSP60/GroE, and low molecular weight families, including Heat Shock Protein 20 (HSP20) or small heat shock proteins (sHSPs) of 15–42 kDa [12].

The HSP20 proteins are ATP-independent molecular chaperones that usually form oligomeric protein complexes ranging from 9 to 50 subunits (200–800 kDa) and act by avoiding protein denaturation in both eukaryotic and prokaryotic cells [16,17]. These chaperones can also assist other chaperones in helping to maintain the native conformation of nascent polypeptide chains and in reorganizing denatured proteins to their native conformation. The main characteristic of HSP20 proteins is a highly conserved 80–100 amino acid sequence referred to as the alpha crystallin domain (ACD) located in the protein’s C-terminal region. This domain is divided into two regions, N-terminal consensus I (27 amino acids) and C-terminal consensus II (29 amino acids), which are separated by a hydrophobic region of variable length. Moreover, the region upstream of the *Hsp*20 coding sequence generally contains several repetitions of the 5′-nGAAnnTTCnnGAAn-3′ (heat shock element (HSE)) sequence, which is recognized and activated by specific transcription factors, designated heat shock factors (HSFs) [12].

Plants have approximately four times more *Hsp*20 genes than animals [18]. This genic and functional diversification could be a consequence of their sessile biology. These proteins are encoded by nuclear multigenic families and are located in different cellular compartments [18]. *Arabidopsis* has 19 genes encoding *Hsp*20, grouped into 12 subfamilies based on their subcellular localization and homology, while 36 *Hsp*20 genes have been described in *Populus trichocarpa* and 23 in *Oryza sativa*[12,19-21]. Other subfamilies have previously been described in other species, totaling 16 subfamilies in plants [19-22].

Recently, genetic evidence has revealed that chaperones play a fundamental role in plant immunity [23]. The chaperone activity of heat shock proteins during biotic stress has been shown to be important for the stability and accumulation of resistance proteins (R proteins) and for the coordination of the entire defense signaling cascade [24]. Thus, HSP20 activity is especially important in crops such as soybean, which are cultivated in large areas around the world and constantly subjected to severe and variable stress conditions. Moreover, soybean is one of the most important crops for providing both animal feed protein and human cooking oil [25,26] and has an important impact on the Brazilian economy [27]. However, nothing is currently known about the *Glycine max* Heat Shock Protein 20 (Gm*Hsp*20) family, and only one *Hsp*20 gene that is responsive to biotic stress has been identified in soybean. This gene was mapped to a Quantitative trait locus (QTL) responsible for *Meloidogyne javanica* resistance and found to be differentially expressed between resistant and susceptible soybean genotypes [3,7].

Given the evidence regarding plant HSP20s and their functional diversification, these proteins are considered ideal targets for improving the development of new varieties of soybean that are tolerant to a wide range of stress conditions or combinations of these stresses. Thus, the main objectives of this study were to identify Gm*Hsp*20 gene family members and carry out their molecular characterization, focusing on the regulation of their expression under different biotic and abiotic conditions, genome distribution and putative promoter structure.

## Results

### Identification and classification of the soybean *Hsp*20 gene family

**Figure 1:**
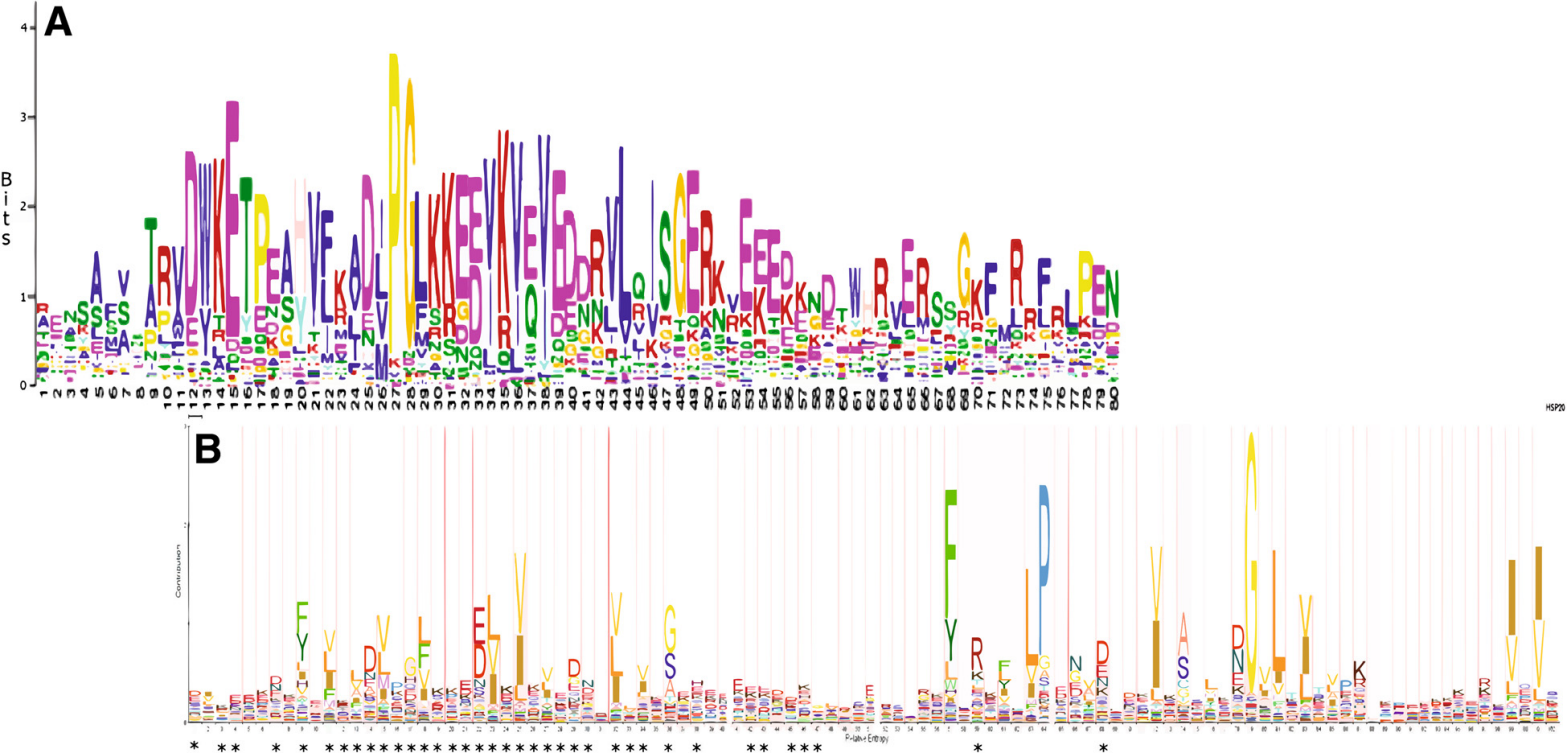
**Alignment of the ACD from the MEME results for the Gm*Hsp*20 with *Hsp*20 from Pfam.** The HMM obtained via MEME analysis for putative Gm*Hsp*20s **(A)**; HMM logo from Pfam representing the *Hsp*20 domain(PF00011). **(B)**. The asterisks indicate matches between HMMs.

Hidden Markov model (HMM) analysis and name search resulted in the identification of 73 and 74 gene models from the Superfamily 1.75 and Phytozome v8.0 Soybean databases, respectively. After removing overlapping hits, 76 putative Gm*Hsp*20 were retrieved. PROSITE and MEME scans of these sequences confirmed the presence of an ACD in 74 of the 76 gene models. However, these ACD were identified in the C-terminal region of only 62 of the putative Gm*Hsp*20 gene models (Figure 1 and Additional file 1: Figure S1).

Three of the putative Gm*Hsp*20 with an irregular ACD disposition (Gm*Hsp*15.2, Gm*Hsp*15.4 and Gm*Hsp*16.2B) were also considered potential Gm*Hsp*20 members because their induction under HS has been previously observed (Additional file 1: Figure S2) [28]. The other 9 candidates eliminated from the analysis due to the position of the ACD and their absence in previous expression studies available in the expression databases. Besides, the Blastp analyses to these first 11 excluded genes also showed that seven genes were similar to unknown or not characterized proteins. One gene showed similarity to a predicted tropinone reductase from soybean. The remaining genes showed a low identity to plant Hsp20 genes.

**Figure 2:**
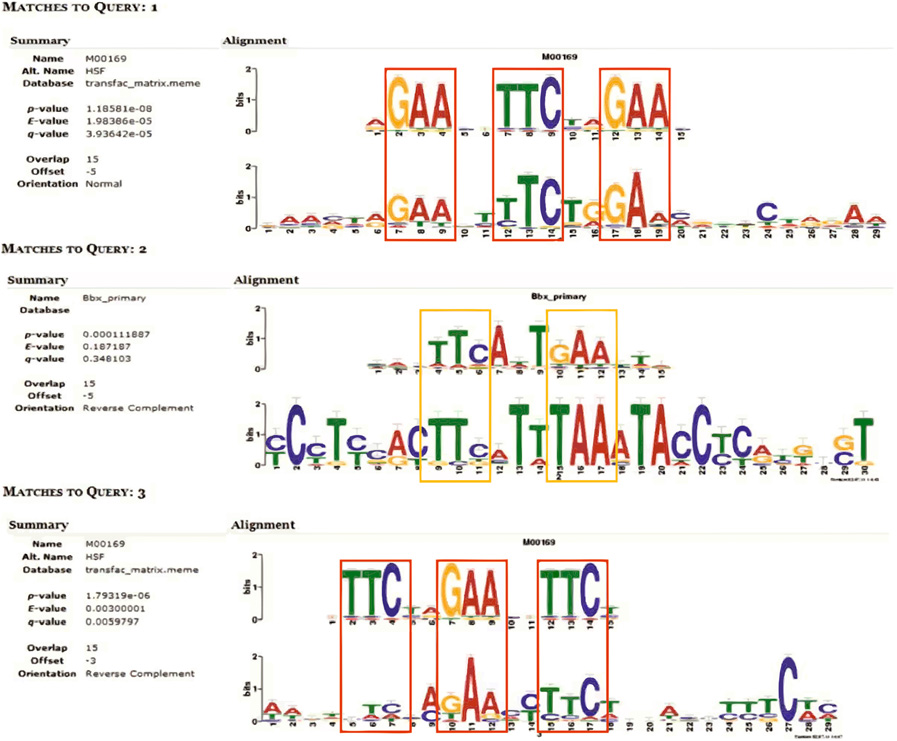
**Hidden Markov model logos obtained using MEME/TOMTOM based on predicted soybean *Hsp*20 promoter sequences.** The motifs obtained via MEME analysis were plotted according to their position within the consensus sequences, and their locations are presented as graphs using HMM. The events are classified by *p*-value and aligned with motifs available at DB. The motif *e*-value is an estimate of the expected number of motifs with the same size and occurrence that would be present in a set of similarly sized random sequences. The heights of the symbols at each motif position indicate sequence conservation. The sequences were manually highlighted to indicate the perfect recognized HSE consensus (red box; nGAAnnTTCnnGAAn or nTTCnnGAAnnTTCn) and the imperfect HSE module (yellow box). Three motifs are found, with the elements being represented by the perfect HSE in alignments 1 (Matches to Query: 1) and 3 (Matches to Query: 3) in the figure. Only the upstream sequences of gene models with a predicted 5′UTR were analyzed.

Using the set of 65 Gm*Hsp*20 candidates, we searched for those candidates that had been detected in previous general gene expression experiments (see Methods). This search resulted in a total of 51 likely candidates (Additional file 2: Table S1). All 51 Gm*Hsp*20 candidates showed at least one repetition of the putative HSE in the 500bp, or 1,500 bp its applied, promoter region (Figure 2; Additional file 1: Figure S3 through S5, and Additional file 2: Table S2). Thus, all of these potential candidates were considered *in silico-*predicted Gm*Hsp*20 genes.

### Complexity and organellar localization of the soybean *Hsp*20 genes

**Figure 3:**
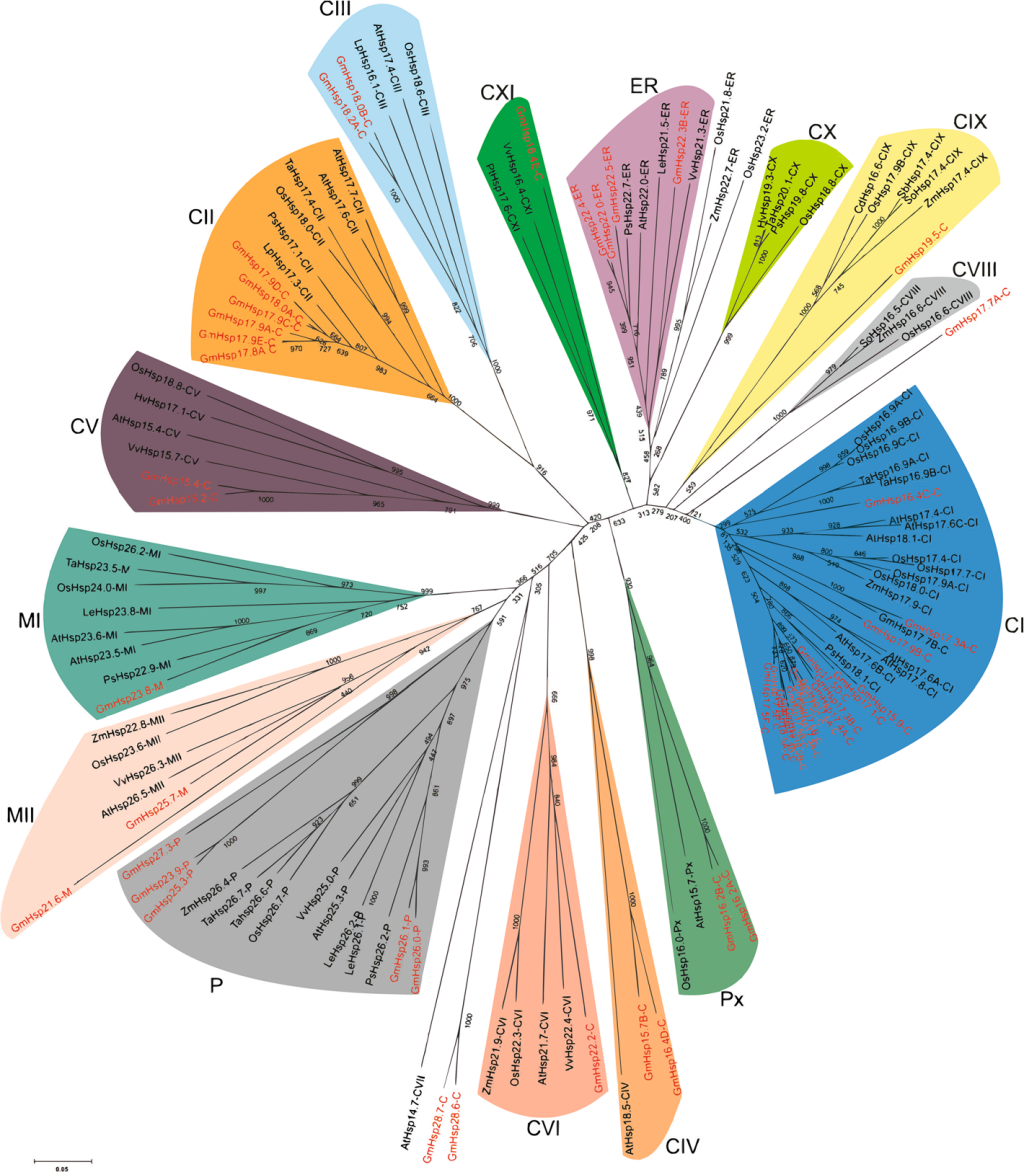
**The unrooted phylogenetic tree for the 51 predicted Gm*Hsp*20 members.** The tree was constructed through alignment of the ACD amino acid sequences from *Hsp*20s from the following species: *Glycine max* (soybean *Hsp*20 genes are highlighted in red); At, *Arabidopsis thaliana*; Os, *Oryza sativa*; Vv, *Vitis vinífera*; Ta, *Triticuma estivum*; Ps, *Pisum sativu*; Le, *Solanum lycopersicum*; Zm, *Zea mays*; Lp, *Lycopersicon peruvianum*; Cd, *Cynodon dactylon*; So, *Saccharum officinarum*; Sb, *Sorghum bicolor*; Hv, *Hordeum vulgare*. The predicted Gm*Hsp*20 genes were attributed to 12 of the 16 known *Hsp*20 subfamilies. ER (endoplasmic reticulum), P (plastid), Px (peroxisome), MI and MII (mitochondrion).

A phylogenetic tree constructed via alignment of the ACD amino acid sequence of the 51 *in silico-*identified Gm*Hsp*20 candidates made it possible to divide them into 13 of the 16 described *Hsp*20 subfamilies (Figure 3). Based on the phylogenetic tree and *in silico* subcellular localization analysis, we identified soybean *Hsp*20 members related to the previously defined CI, CII, CIII, CIV, MI, P, ER and Px subfamilies as well as to the recently identified CV, CVI, CIX, CXI and MII subfamilies [12,18,22]. In addition, we identified three orphan genes, two of which (Gm*Hsp*28.6 and Gm*Hsp*28.7) clustered with the *Arabidopsis* At*Hsp*14-7 CVII subfamily and were found to be heat responsive in our *in vivo* analysis; however, the cluster bootstrap value was low (305; threshold > 500). The third orphan gene was Gm*Hsp*17.7A (not heat responsive).

Thus, the 51 Gm*Hsp*20 genes were distributed among a total of 15 subfamilies as follows: 37 were nucleo-cytoplasmic (C) *Hsp*20 genes (eight subfamilies and three orphan genes); three were mitochondrial (M) *Hsp*20 genes (two subfamilies); four were endoplasmic reticulum (ER) *Hsp*20 genes, five were plastidial (P) *Hsp*20 genes and two were peroxisomal (Px) *Hsp*20 genes.

**Figure 4:**
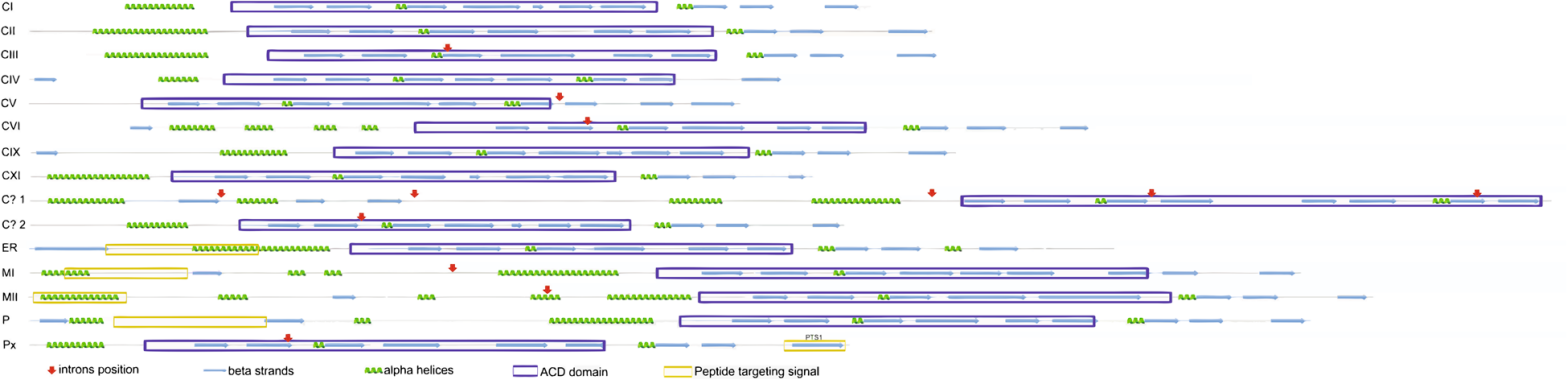
**Diagram illustrating the patterns identified for the predicted secondary structures of the Gm*HSP*20 subfamilies.** The positions of introns are indicated with red arrows. The blue horizontal arrows delimit the regions of beta sheet formation. Spirals in green delimit the regions of possible α-helix secondary structures. The blue and yellow rectangles delimit the regions predicted to constitute the ACD and signal peptides, respectively.

In addition to phylogenetic analysis and prediction of subcellular localization, the prediction of protein secondary structure models for GmHSP20 subfamilies is important for determining the subfamily distribution (Additional file 2: Table S3). Subfamilies CI and CII contain amino terminal α-helices and a variable number of β-sheet segments, seven segments in CI members and six in CII members (Figure 4). Subfamily CIII is very similar to CII, except that all members of the CIII subfamily exhibit one intron in the ACD and another in the third β-sheet segment. In addition, the CIII Gm*Hsp*20 genes present an intron in the 5′UTR region. Cytoplasmic subfamily CIV contains seven β-sheet segments and two α-helices in the ACD. Subfamily CV exhibits a conserved pattern in relation to the secondary structure observed in rice and *Arabidopsis*, where the presence of an intronic region just after the second β-sheet in the ACD is a peculiar feature.

The most important characteristic of the secondary structure of the ER Gm*Hsp*20 subfamily is the presence of two large β-sheets and an α-helices in the N-terminal region, where a signal peptide was also predicted. The two mitochondrial subfamilies contain four to five α-helices and one β-sheet in a small segment of the N-terminal region. In MI subfamily members, seven small β-sheet segments were identified, while only five segments were identified in the MII subfamily members. The profiles of MI and MII regarding the position of the ACD in the C-terminal region and the occurrence of an intron in the center of the protein are unique among the Gm*Hsp*20 families. A diagrammatic representation of the *Hsp*20 subfamilies showing the ACD region, intron positions, transit peptides and secondary structures is presented in Figure 4.

An amino acid sequence alignment of the 51 Gm*Hsp*20 proteins showed that the identity among the sequences varies from 17.50% to 98.99%. The highest values were detected in the C-terminal region corresponding to the ACD, while the lowest values were observed between members of different subfamilies.

The motive analysis of the *Hsp* candidates reveled that among the 51 possible Gm*Hsp*20 genes, 33 were intronless, while 11 contain only one intron, and seven showed exhibit two introns or more. The intron occurrences were validated in two gene models using conventional PCR. DNA amplification of Gm*Acd*33.0 and Gm*Acd*23.1 confirmed the presence of intron fragments of 527 and 457 bp, respectively, because cDNA amplification produced the expected amplicon as predicted *via* genome annotation using the Phytozome database (Additional file 1: Figure S6).

The predicted molecular weights of the GmHSP20 candidates were distributed in a range from 15.24 kDa (Gm*Hsp*15.2, 134 aa) to 28.71 kDa (Gm*Hsp*28.7, 262 aa). The predicted isoelectric points of the GmHSP20 candidates were between 5.11 (Gm*Hsp*16.4D) and 9.52 (Gm*Hsp*25.3). Interestingly, the predicted instability indices showed that only 10 of the 51 GmHSP20 candidates could be considered stable proteins (cutoff ≤40) (Additional file 2: Table S4).

### Genome organization and gene duplication

The 51 putative *Hsp*20 gene candidates are distributed across 17 of the 20 chromosomes in the soybean genome. No Gm*Hsp*20 genes were detected on chromosome 3, 5 or 9. Interestingly, closely related sequences of the CI subfamily clustered together in the phylogenetic tree and are mainly located on chromosomes 7 and 13, suggesting that the expansion of this gene family may have occurred via localized or intra-chromosomal duplication.

**Figure 5:**
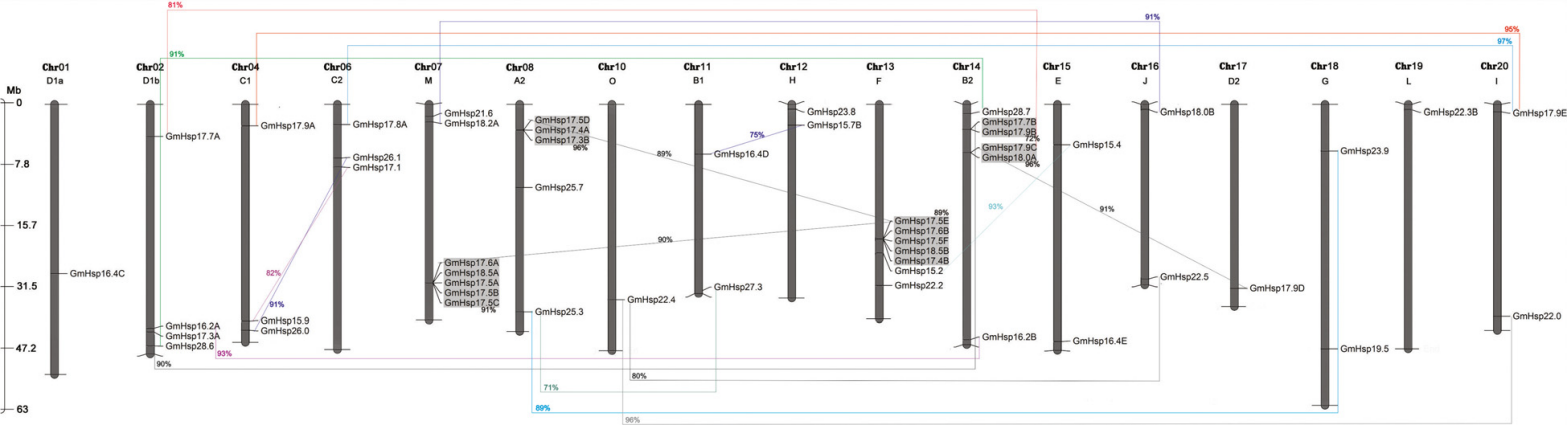
**Locus map and duplication event coordinates of paralogous Gm*Hsp*20 gene candidates.** The identity of each linkage group is indicated at the top of each bar. Only the chromosomes where Gm*Hsp*20 genes were mapped are shown. Possible duplicated genes are connected by lines, and the percentage of identity is annotated in the same stroke color. Gray rectangles indicate segmental duplications. The bar located on the left side indicates chromosome sizes in megabases.

Four *Hsp*20 paralog gene groups were identified on chromosomes 14, 2, 4 and 17. Furthermore, duplication with a high similarity (96%) was detected between Gm*Hsp*22.4 in the terminal region of chromosome 10 and Gm*Hsp*22.0 at the same region of chromosome 20. Gm*Hsp*22.0 also shared a similarity of 80% with Gm*Hsp*22.5 (Additional file 3: Table S6). Gm*Hsp*17.9E on chromosome 20 also showed a likely (97%) duplicated region shared with Gm*Hsp*17.8A on chromosome 6, both of which are located on the upper arm of chromosomes. Finally, chromosomes 4 and 6 contain three putative duplications, one presenting 82% similarity, involving two of the four genes classified in subfamily P. The duplication prediction analysis indicated that the evolution of the *Hsp*20 gene family in the soybean genome resulted from a total of 23 gene duplications, five of which were segmental among four chromosomes (Figure 5).

### Gm*Hsp*20 expression under heat shock and cold treatments

To validate the 51 putative Gm*Hsp*20 candidates under stress, we investigated their *in vivo* expression profiles in heat shock and cold experiments. Out of the 51 primers designed for these individual Gm*Hsp*20 genes, 43 produced only one amplicon and were used in this analysis. In addition, two gene models resulting from the database exploration analyses that showed a high homology with rice *Acd* genes were also analyzed.

**Figure 6:**
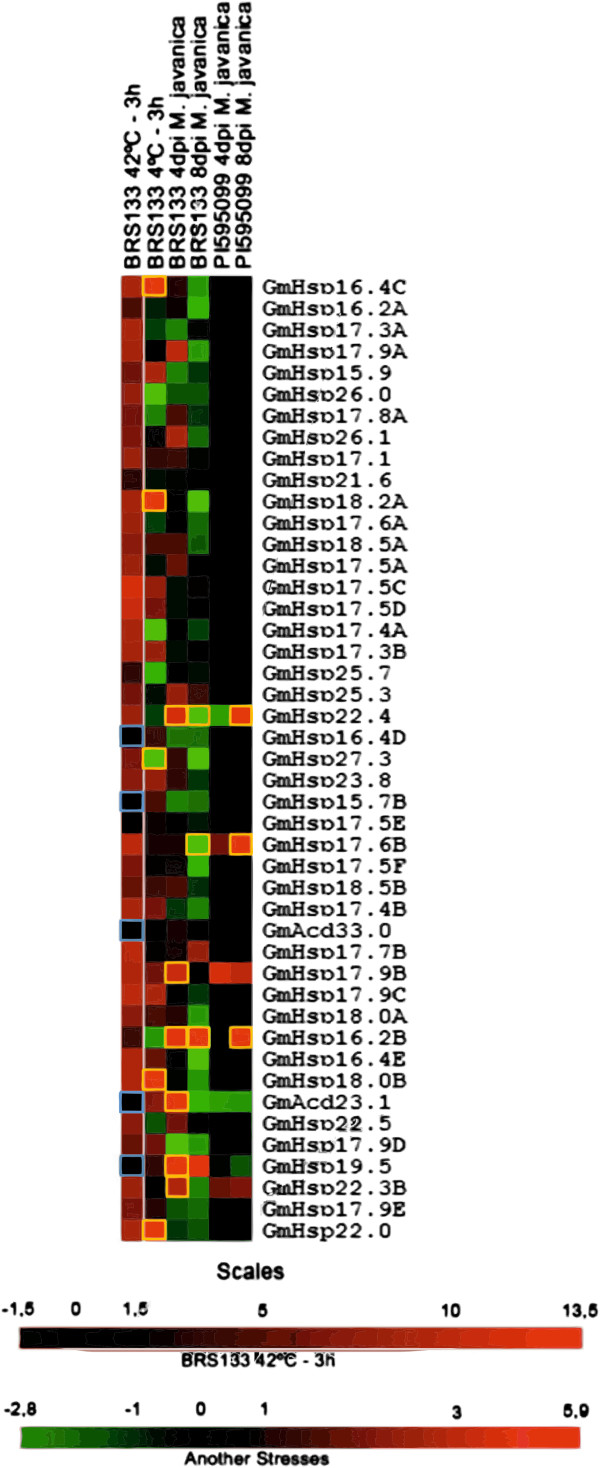
**Heat map of the expression profiles of 43 Gm*Hsp*20 candidates and 2 *Acd *genes.** The expression profiles were analyzed under biotic (nematode infection) and abiotic (heat and cold) stress conditions. The expression profiles under stress conditions, based on qRT-PCR data, are presented as heat maps generated using TreeView 1.60 software. The transcript levels following heat shock stress are depicted using a color scale indicating log10 values and are shown beside the transcript levels following the other stresses. The spots highlighted in yellow indicate the genes that showed a significant expression level change (at a 5% significance level) compared with the control under cold and biotic stresses treatments. The spots highlighted in blue indicate the genes that did not exhibit a significant expression level change (at a 5% significance level) compared with the control under heat shock treatment.

Among the 43 Gm*Hsp*20 candidates analyzed *in vivo,* 40 showed strong induction under heat shock, which was easily visualized using conventional PCR (Additional file 1: Figure S7). The expression of all candidates under heat shock treatment was quantified via quantitative real-time polymerase chain reaction (qRT-PCR) (Figure 6 and Additional file 2: Table S5). Based on the obtained results, four of the Gm*Hsp*20 candidates (Gm*Hsp*16.4D, Gm*Hsp*15.7B, Gm*Hsp*17.7A and Gm*Hsp*19.5) and two *Acd* genes (Gm*Acd*33.0, Gm*Acd*23.1) were not induced significantly in stressed soybean roots compared with the control conditions. To check whether these gene models were induced in another part of the plant exposed to heat shock, we also performed a qRT-PCR analysis using foliar samples, but no induction was detected.

As expected, cold stress showed a weaker influence on Gm*Hsp*20 expression compared with the heat treatment. Under cold conditions, only five genes were responsive. Gm*Hsp*18.2A, Gm*Hsp*18.0B, Gm*Hsp*16.4C and Gm*Hsp*22.0 were induced, while Gm*Hsp*27.3 was down regulated; the first two genes belong to CIII and the others to the CI, ER and P subfamilies, respectively. All five genes were also induced under the heat stress treatment.

### Gm*Hsp*20 expression under *M. javanica* infection and differences in resistant and susceptible soybean genotypes

The expression of the 51 putative Gm*Hsp*20 candidates was also monitored in soybean plants inoculated with *M. javanica* as a biotic stress model. The qRT-PCR analysis following the biotic stress treatments resulted in the identification of six responsive Gm*Hsp*20 genes and one Gm*Acd* gene (Gm*Acd*23.1). Five of the six Gm*Hsp*20 genes induced by heat shock stress, Gm*Hsp*22.4, Gm*Hsp*17.6B, Gm*Hsp*17.9B, Gm*Hsp*16.2B and Gm*Hsp*22.3B, were also significantly induced in at least one of the biotic stress treatments tested, while the other *Hsp*20 gene, Gm*Hsp*19.5, exhibited induction that was detectable only at 4 days post-inoculation (dpi) and was not responsive to heat shock stress. Gm*Hsp*22.4 was induced at 4 dpi and repressed at 8 dpi in BRS 133, while Gm*Hsp*17.6B was repressed at 4 dpi (Figure 6). Four genes, Gm*Hsp*17.9B, Gm*Hsp*19.5, Gm*Hsp*22.3B and Gm*Acd*23.1, were induced only at 4 dpi in BRS 133, while Gm*Hsp*16.2B was induced at both 4 dpi and 8 dpi.

**Figure 7:**
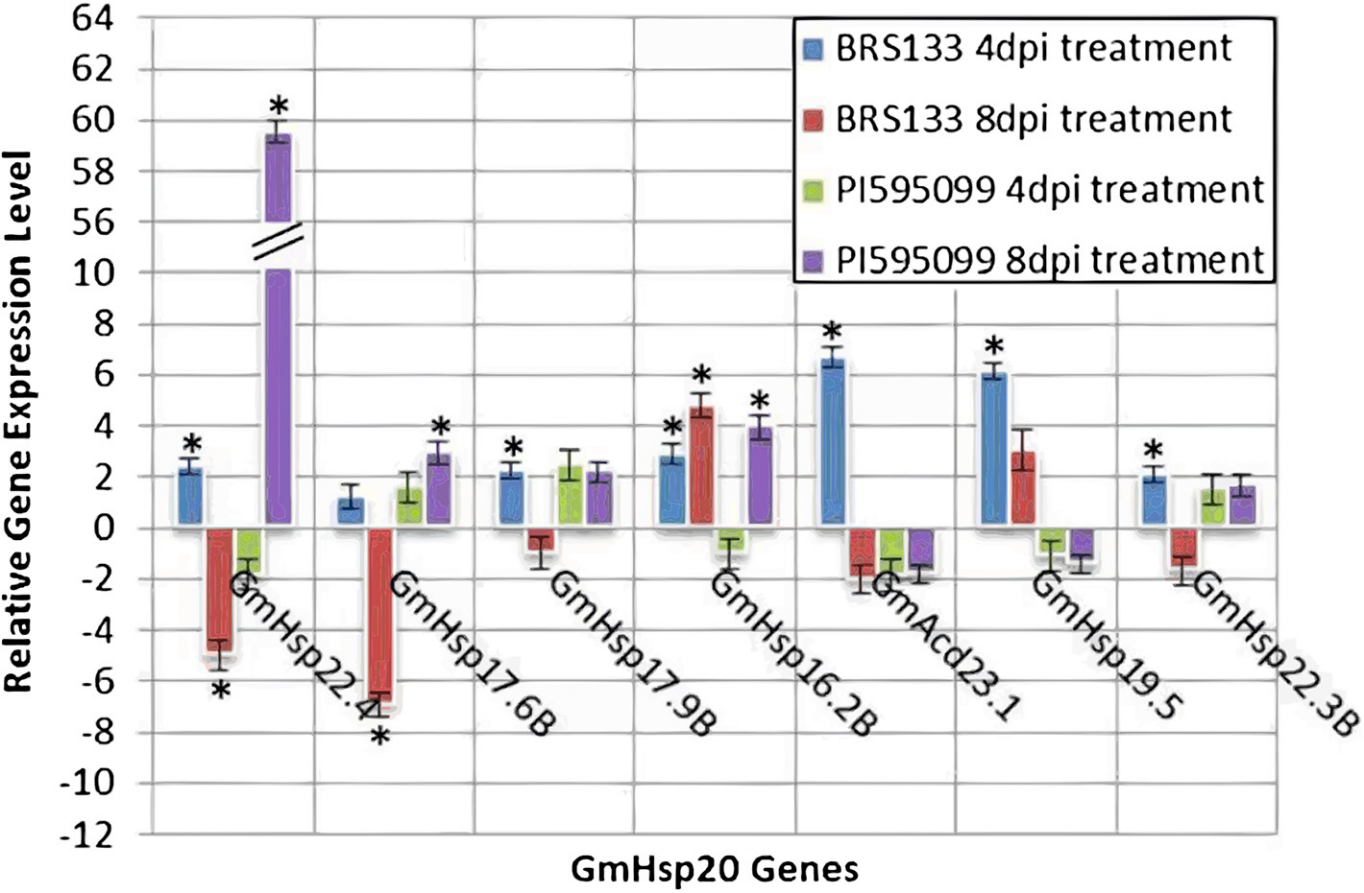
**Expression profile under the stress of *M. javanica *infection in resistant and susceptible soybean genotypes.** The relative expression results obtained via qRT-PCR for the Gm*Hsp*20 candidates evaluated in the resistant (PI 595099) soybean genotype under *M. javanica* infection (4 and 8 dpi). Error bars indicate the margin of error.

When the nematode-induced Gm*Hsp*20 genes were compared between the two soybean genotypes, four gene models showed a differential expression profile: Gm*Hsp*16.2B, Gm*Hsp*22.3B, Gm*Hsp*17.6B and Gm*Hsp*22.4. Gm*Hsp*16.2B and Gm*Hsp*22.3B were induced in the susceptible genotype (BRS 133): the former at both 4 and 8 dpi, and the latter only at 4 dpi. Interestingly, both Gm*Hsp*22.4 and Gm*Hsp*17.6B were down-regulated in BRS 133 at 8 dpi and up-regulated in the resistant genotype (PI 595099) at 8 dpi (Figure 7). The complete arrangement of the genes according to their expression profiles after the treatments can be seen in the Venn diagram provided as Additional file 1: Figure S8.

The analysis of disease resistance QTL locations in the soybean genome using data on corresponding molecular markers (http://soybase.org/) confirmed a strong bond between these sites and the *Hsp*20 genes. At least, one QTL appears to be related to 22 of the 51 Gm*Hsp*20 gene candidates (Additional file 4: Table S7). Among the QTLs reported in SoyBase that are related to nematode and fungal disease resistance, 45 QTLs were found to be physically located near to, in approximately 2 Mb flanking regions, at least one Gm*Hsp*20. The Gm*Hsp*17.9A gene, reported by Kandoth et al. [6] to be related to *Heterodera glycines* infection, was identified as being located near a QTL for resistance to such infection (SCN 18–3). The Gm*Hsp*17.4A, Gm*Hsp*22.4 and Gm*Hsp*17.6B genes are also situated near QTLs involved in biotic stress.

### Characterization of putative *cis*-elements in Gm*Hsp*20 promoters induced by abiotic and biotic stresses

The 51 *Hsp*20 candidates were also evaluated regarding the occurrence and distribution of putative *cis*-elements in their promoter regions. For this analysis, 48 of the 51 *in silico*-predicted candidates, plus two Gm*Acd* genes, were considered based on the availability of promoter regions in Phytozome (see Methods – predicted 5′UTR). The promoter regions exhibit a characteristic consensus TATA box, which was identified in 29 members, followed by putative HSEs that are present in all 50 genes, ranging from one to four repeats (Table 1). The putative HSEs are mostly concentrated approximately 150 bp upstream of the transcription start site. Exceptions to this pattern were found (Gm*Hsp*16.2A, Gm*Hsp*15.2 and Gm*Hsp*15.4) in which a single putative HSE was identified, occurring approximately 500 bp or more upstream (up to 1,500 bp upstream).

**Table 1:** ***Cis*-elements in the Gm *Hsp *20 promoters – their occurrence and position**

**MatInspector – PLACE – PlantCARE**				
**Gm *Hsp *20 gene candidates**	^**a**^**CCAAT**	^**a**^**TATA box**	^**a**^**W-box**	**TA-rich region**
Gm*Hsp*16.4C	−14 -110 -280 -333 -377	−29	____	____
Gm*Hsp*17.7A	____	−44	____	____
Gm*Hsp*16.2A	−2 -105 -165 -166 -262 -356 -471 -472	____	−17 -323	____
Gm*Hsp*17.3A	−86 -158 - 290–299 - 447–468 -473	−30	−263 -264	____
Gm*Hsp*28.6	−676	____	−66	____
Gm*Hsp*17.9A	−372	____	____	−309
Gm*Hsp*15.9	−119 -120 -300 -488	____	____	____
Gm*Hsp*26.0	−296 -395	−32	____	____
Gm*Hsp*17.8A	−196 -253 -290 -409	−32	−120	____
Gm*Hsp*26.1	−17 -186	−28	−5	____
Gm*Hsp*17.1	−154 -229 -357	____	−41 -170 -169 -396	____
Gm*Hsp*21.6	n/a	n/a	n/a	n/a
Gm*Hsp*18.2A	−36- 67–306 -330 -406	−19	−315 -165 -493	____
Gm*Hsp*17.6A	−178 -301	____	____	____
Gm*Hsp*18.5A	−442	−25	____	____
Gm*Hsp*17.5A	−401 -202	____	____	____
Gm*Hsp*17.5B	n/a	n/a	n/a	n/a
Gm*Hsp*17.5C	−313	____	−215	____
Gm*Hsp*17.5D	−491	−28	−306	
Gm*Hsp*17.4A	−188 -219 -238 -275 -312	____	____	____
Gm*Hsp*17.3B	−85 -106 -116 -135- 249 -347	____	____	____
Gm*Hsp*25.7	−13 -284	−40	−87	____
Gm*Hsp*25.3	−1 -40 -91	−29	−496	____
Gm*Hsp*22.4	−253 -370	−19	−406	−4
Gm*Hsp*16.4D	−42 -103 -247 -278 -425 -440	−8	____	____
Gm*Hsp*27.3	−109	−27	−96	____
Gm*Hsp*23.8	−705	____	−69 -292	____
Gm*Hsp*15.7B	−28 -130 -159	____	____	____
Gm*Hsp*17.5E	−215 -304 -494	−26	____	____
Gm*Hsp*17.6B	−69 -420	____	−4	−178
Gm*Hsp*17.5F	−246 -333 -461	−57	−481 -377	____
Gm*Hsp*18.5B	−260	−24	−331 -437 -464	____
Gm*Hsp*17.4B	−633	____	−198 -342 -398	____
Gm*Hsp*15.2	−725	−24	−442	____
Gm*Hsp*22.2	−326 -397	____	−169 -252 -366	____
Gm*Hsp*17.7B	−176 -218 -377 -493	−26	−288	____
Gm*Hsp*17.9B	−56 -74 -238	−44	−71	−44
Gm*Hsp*17.9C	−952	−32	−240 -390	____
Gm*Hsp*18.0A	−1402	____	−167	____
Gm*Hsp*16.2B	−77 -198 -472	−25	−476	____
Gm*Hsp*15.4	−474	____	____	____
Gm*Hsp*16.4E	____	____	____	____
Gm*Hsp*18.0B	−72 -76 -442	−24	____	____
Gm*Hsp*22.5	−271 -396	−26	____	____
Gm*Hsp*17.9D	−128 -301 -331	−29	−222	____
Gm*Hsp*23.9	n/a	n/a	n/a	n/a
Gm*Hsp*19.5	−50 -157 -293 -300 -341 -452	−35	____	____
Gm*Hsp*22.3B	−103 -221 -308 -320	____	____	____
Gm*Hsp*17.9E	−207 -265 -345 -398 -433	____	−131 -477	____
Gm*Hsp*22.0	____	____	−21	____
Gm*Hsp*28.7	−191 -217 -302 -329	−42	−97 -100 -336	____
**Gm *Acd *33.0**	−46 -134 -252 -256 -454 -474 -488	−34	−533	____
**Gm *Acd *23.1**	−22 -88 -294	−28	−412 -452	−251

The CCAAT, TATA box, W-box and TA-rich positions identified 500 bp upstream of the Gm*Hsp*20 candidates are estimated in relation to the transcription start site. ^a^ The results are presented according to MEME, MatInspector and Place software. The TA-rich elements were obtained using the program PlantCare. The abbreviation “n/a” means that the upstream region is not available on Phytozome.

Other elements known to function as *cis*-elements in *Hsp*20 genes, such as CAAT box, W-box and TA-rich regions, were also predicted at variable positions [7,14,29]. A putative CAAT box was detected in 47 genes and a putative W-box in 31. Among the Gm*Hsp*20 genes identified in this study as being responsive to nematode infection, four showed at least one putative W-box site: Gm*Hsp*22.4, Gm*Hsp*17.6B, Gm*Hsp*17.9B and Gm*Hsp*16.2B. All four of these genes were induced by high temperature (HT).

A putative TA-rich region was present at different positions, especially in the promoters of the *Hsp*20 genes induced by biotic stress. These regions were found 309, 178, 4, 44 and 251 bp upstream of the transcription start site of the genes Gm*Hsp*17.9A ((TA)_12_), Gm*Hsp*17.6B ((TA)_9_), Gm*Hsp*22.4 ((TA)_13_), Gm*Hsp*17.9B ((TA)_6_) and Gm*Acd*23.1 ((TA)_8_), respectively. Three of these genes, Gm*Hsp*17.6B, Gm*Hsp*22.4 and Gm*Hsp*17.9B, were induced in soybean roots after heat stress or infection with *M. javanica* in our experiments based on qRT-PCR analysis. Gm*Hsp*17.9A was not induced in our biotic experiments but has previously been described as being responsive to *H. glycines*[6].

**Figure 8:**
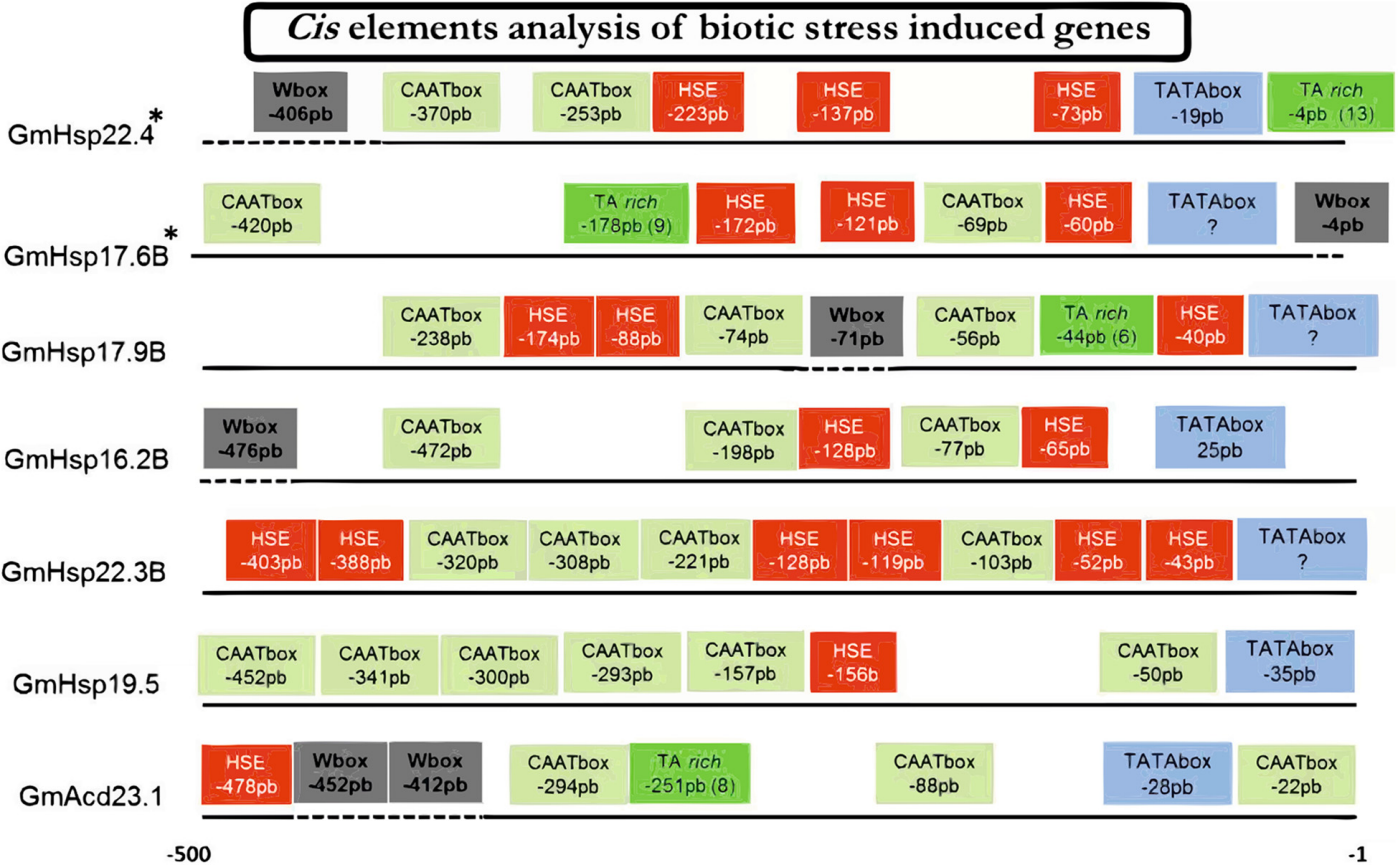
***Cis-*element analysis in the biotic stress-induced genes.** Place, Genomatix, MEME and PlantCare were used to analyze the region 500 bp upstream of each of the seven genes that were responsive to biotic stress (six Gm*Hsp*20 genes and one Gm*Acd*). Blue boxes indicate TATAbox elements; red, the HSE; gray-green, the CAAT boxes; green the TA-rich elements; and gray, the W-box elements. Genes Gm*Acd*23.1 and Gm*Hsp*19.5 were responsive to only biotic stresses, while the others were also heat-responsive.

Overall, it was possible to identify an organization pattern of the putative *cis*-elements in the promoters of the six Gm*Hsp*20 candidates that were responsive to nematode infection. CAAT boxes presented a heterogeneous distribution ranging from two to five repetitions within the region containing putative HSEs or immediately upstream, while putative W-boxes were located more distantly, upstream of the putative HSEs. In all six Gm*Hsp*20 genes, the first putative HSE is located within the first −83 bp upstream of the start site, while the putative HSE was followed by at least one putative CAAT box. At least one putative W-box was identified in four of the six Gm*Hsp*20 genes (Gm*Hsp*22.4, Gm*Hsp*17.6B, Gm*Hsp*17.9B and Gm*Hsp*16.2B) induced by nematodes, but they were all at different positions (−406 bp, -4 bp, -71 bp and −476 bp, respectively) (Figure 8).

### Gm*Hsp*20 putative operational promoter models

**Figure 9:**
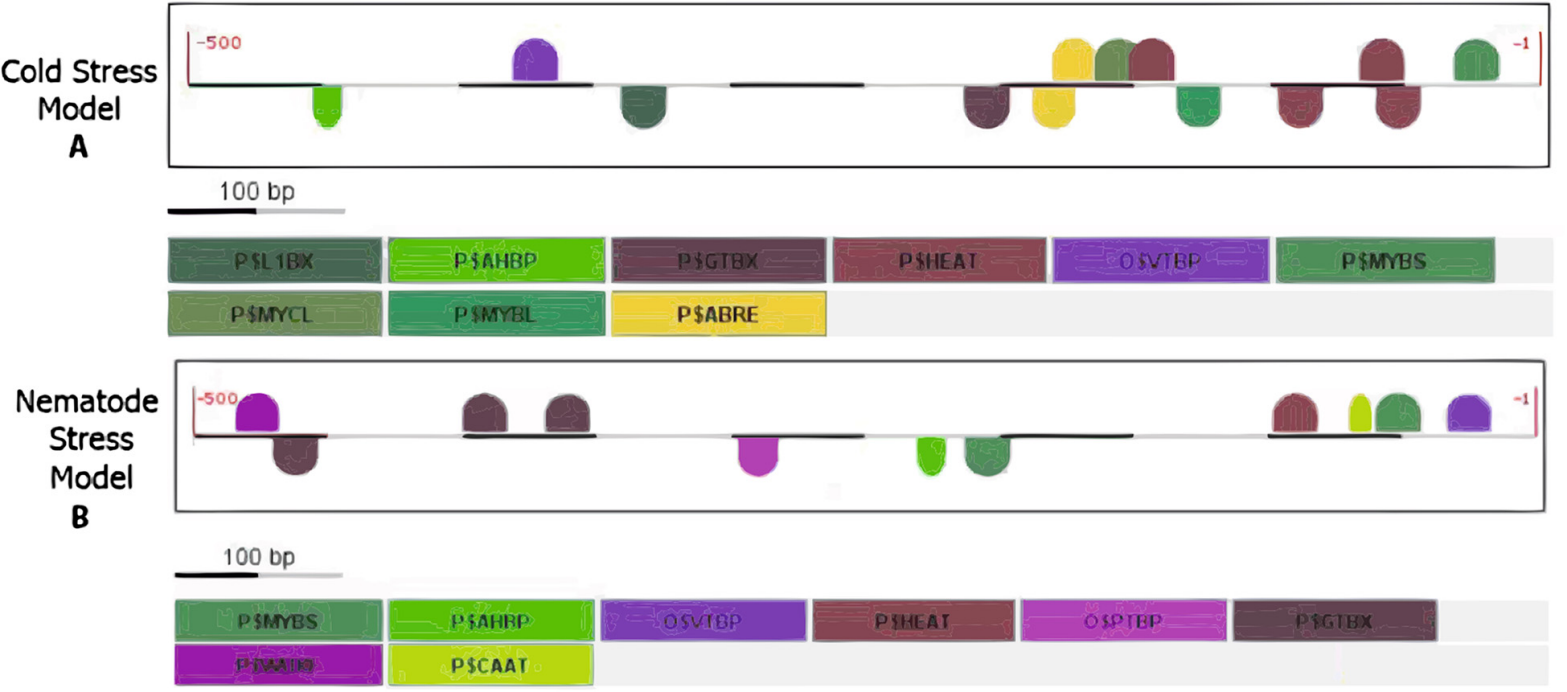
**Illustration of the elements of the structural models of the Gm*Hsp*20 gene promoter region.** The Cold Stress and Nematode Stress Models were identified using Frameworker/Genomatix. This program was employed to search for the main common elements between genes and the highest frequencies of their positions. In the Cold Stress Model **(A)**, the identified HSEs positioned with a negative orientation have *p*-values of 0.0381311. The model for the AHBP, GTBX, VTBP (TATAbox vertebrate) and PTBT (TATAbox plant) elements has a *p*-value 1.67 e^-04^. The model with a W-box shows a *p*-value of 5.02 e^-01^. Another model identified a CAAT box with a *p*-value of 0.167564. In the Nematode Stress Model **(B)**, the identified HSE position has a *p*-value of 9.37 e^-01^. The model with the ABRE, MYBS, MYCL and HEAT *cis*-elements presented a *p*-value of 9.57 e^-04^. Another model, without stabilization of the mandatory elements, included the L1BX, AHBP, GTBX, VTBP and HEAT elements with a *p*-value of 4.789 e^-06^. All of the models found for each specific stress were concatenated to form a single representative model.

The results of the *in silico* analysis and *in vivo* expression profiling of the Gm*Hsp*20 candidates under biotic and abiotic stress conditions were used to determine a putative transcription factor binding site (TFBS) combinatorial models for their promoters. Comparative analysis of the promoters of the five Gm*Hsp*20 genes responsive to cold stress (Gm*Hsp*16.4C, Gm*Hsp*18.2A, Gm*Hsp*18.0B, Gm*Hsp*27.3 and Gm*Hsp*22.0) resulted in five putative operational models containing the mandatory HEAT element. Other putative common elements were L1BX, AHBP, GTBX, VTBP, MYBS, MYCL, MYBL and ABRE (Figure 9).

Analysis of the promoters of the six gene candidates induced by biotic stress (*M. javanica*), Gm*Hsp*22.4, Gm*Hsp*17.6B, Gm*Hsp*17.9B, Gm*Hsp*16.2B, Gm*Hsp*22.3B and Gm*Hsp*19.5, resulted in the identification of three putative models, all of which included the mandatory putative HEAT element and the putative elements AHBP, GTBX, VTBP, MYBS, WBXF and CAAT.

## Discussion

Based on a genome-wide analysis, we propose that the soybean genome is enriched in small heat shock genes, presenting 51 *Hsp*20 genes, compared with the 31 and 39 *Hsp*20 genes observed in *Arabidopsis* and rice, respectively [12,21,30]. This greater expansion of the *Hsp*20 gene family in soybean was expected because the soybean genome has experienced successive duplications throughout its evolution [25]. According to our analysis, a total of 23 gene duplications of the *Hsp*20 family can be predicted to have occurred in the soybean genome. As reported above, the vast majority of the Gm*Hsp*20 genes (47 gene models) were strongly induced by heat treatment (Figure 6), which suggests that our *in silico* pipeline for predicting Gm*Hsp*20 candidates was efficient. Three of the Gm*Hsp*20 candidates were expressed only under non-stressed conditions, while Gm*Hsp*19.5 was exclusively induced after *M. javanica* inoculation. These results indicate that some Gm*Hsp*20 genes exhibit functions that are unrelated to heat shock under normal growth conditions, for example, specific housekeeping activities, in addition to more specialized activities, such as in the response to biotic stress. This functional diversification of the *Hsp*20 gene family has also been reported in sunflower and rice [12,31].

In earlier attempts to categorize the subfamilies of *Arabidopsis Hsp*20 genes, it was proposed that the majority of the At*Hsp*20 could be divided into seven subfamilies (CI, CII, CIII, M, P, ER and Px) and that five other genes do not fall into any subfamily [21,22]. In a more recent analysis, the At*Hsp*20 gene family was extended to include 12 subfamilies based on placing the five uncategorized *Hsp*20 genes into four new nucleocytoplasmic subfamilies (CIV, CV, CVI and CVII) and adding a new mitochondrial subfamily, MII [22]. A recent categorization of the rice *Hsp*20 gene family proposed a distribution of Os*Hsp*20 genes into 16 subfamilies: four nucleocytoplasmic subfamilies (CVIII, CIX, CX and CXI) plus 12 subfamilies already identified in *Arabidopsis*[12]). Gm*Hsp*20 clustering with *Arabidopsis* subfamily CVII and rice subfamilies CVIII and CX was not observed in the present study when bootstrap values were considered. However, in the 15 remaining subfamilies, we were able to identify at least 51 members, two of which subfamilies have not yet been described in the literature. Our results suggest that there are 10 nucleocytoplasmic subfamilies in soybean, the largest of which is subfamily CI, with 19 members (Figure 3).

The large number of Gm*HSP*20 proteins classified into nucleocytoplasmic subfamilies is a feature shared with other species, such as *Arabidopsis* and rice [12,22,30], and indicates that the cytoplasm may be the primary site of action for *HSP*20 proteins. In the cytoplasm, where protein assembly occurs, a higher concentration of *Hsp*20 proteins could prevent in appropriate folding or interactions that could lead to the formation of prejudicial aggregates.

Notably, in the phylogenetic analysis, the *Hsp*20 genes from different species that are classified in the same subfamily were observed to be more closely related than the members of the same species that belong to different subfamilies. This finding gave us an indication that synteny might exist among soybean, rice and *Arabidopsis HSP*20 proteins. The *Hsp*20 genes most likely had a common ancestor that gave rise to the different subfamilies before the diversification within these species [30].

Three soybean genes (Gm*Hsp*28.7, Gm*Hsp*28.6 and Gm*Hsp*17.7A) were not grouped into any of the known *Hsp*20 subfamilies (Figure 3). Among these so-called orphan genes, Gm*Hsp*17.7A was not responsive to heat shock stress, despite its high similarity with rice *Hsp*23.2-ER, which is HT responsive (Figure 6).

Regarding gene organization, 64% (33 of the 51 gene candidates) of the soybean *Hsp*20 genes are intronless based on genome prediction and qRT-PCR data, which is similar to the percentage reported for rice *Hsp*20s (74%) [12]. Few of the Gm*Hsp*20 genes contain introns, and their lengths are highly variable. The relationship between the occurrence of introns and the expression level of a gene is controversial [32,33]. In some studies, the absence of an intron, or a short intron length, has been found to enhance the level of gene expression in plants [34,35]. In addition, there are indications that during evolution, genes must be rapidly activated in response to stress tend to show a decreased intron density [36]. This may be the mechanism that has led to more rapid induction of the expression of plant *Hsp*20 genes, which occurs within a few minutes after the initiation of heat shock [12].

Among the Gm*Hsp*20 genes containing introns, 10 (35.71%) contain only one intron, and two (Gm*Hsp*18.0B and Gm*Hsp*18.2A) contain an intron in the 5′UTR region; these two genes were induced by cold stress. According to Kamo et al. [37], the presence of an intron in the 5′UTR region can potentiate the translation process.

Furthermore, our results indicate that the Gm*Hsp*20s can be classified as unstable proteins, since 76.5% of aminoacid sequence showed an unstable profile (when instability index threshold were considered [38]) (see Additional file 2: Table S4). An unstable profile is believed to be a common feature among stress-induced proteins [39]. Considering that HSP20 proteins are synthesized at a specific time in the cell, their instability indicates a rapid turnover that should allow transcriptional regulation of these proteins in the cellular environment [31,40].

As expected, the Gm*Hsp*20 genes were preferentially located in terminal regions of the soybean chromosomes, which have been demonstrated to be enriched in genes in the soybean genome [25]. This localization might contribute to the occurrence of segmental duplications in the soybean *Hsp*20 family. Similarly, the genome duplications experienced by the soybean genome during its evolution and the high recombination rates between segmental regions of homologous chromosomes might have increased the occurrence of gene duplications [41] and, consequently, favored the expansion and functional diversification of the Gm*Hsp*20 family. Based on our analysis and the findings of Schmutz et al. [25], particularly their conclusions about soybean genome evolution and organization, we suggest that the evolution of the soybean *Hsp*20 family has involved a total of 23 gene duplications, five of which were segmental on four different chromosomes (Figure 5). The same number of duplications has been reported for the rice genome, in which 23 Os*Hsp*20 genes were originated via duplication events [30]. These segmental duplications appear to have contributed significantly to increasing the number of members of the soybean CI subclass, located on chromosomes 7, 8, 13 and 14. In rice, the CI members are also distributed in clusters of segmental duplications [30].

Considering the concept of parsimony, the conservation of this pattern of *Hsp*20 gene duplication within the same chromosome observed in the genomes of rice, *Arabidopsis* and soybean most likely originated through segmental duplication events that occurred before the divergence of monocots and dicots, in the ancestral species, followed by chromosomal duplications in both the ancestral species and within each species [25,42]. Still, it is notable that three of the six Gm*Hsp*20 genes that are responsive to *M. javanica* and *H. glycines* infection (Gm*Hsp*17.4A, Gm*Hsp*17.9B [6] and Gm*Hsp*17.6B [7]) are organized in blocks of segmental duplications in the genome. In soybean, expansion of the segmental gene families associated with the basal resistance response is common and has been observed in families including NBS-LRR, F-box and auxin-responsive genes [25]. Such duplications may contribute to the diversification of relevant alleles during plant-pathogen interactions or to the maintenance of similar levels of gene expression within the block, as observed in rice [30,43]. However, unlike the results reported by Ouyang et al. [30], the expression pattern of the tandem duplicated genes, under the stress conditions tested here, was observed to be highly heterogeneous for GmHsp20.

Based on the organization of the soybean genome, the number of *Hsp*20 paralog gene groups observed between chromosomes 14, 2, 4 and 17 corroborates their high synteny, as described by Schmutz et al. [25]. Furthermore, this organizational characteristic was observed between chromosomes 20 and 10 as well as between 6 and 4, but 7.08% of the length of chromosome 20 is still homologous to fragments of four other chromosomes [25]. The putative interchromosomal duplication observed between Gm*Hsp*22.4 and Gm*Hsp*22.0, located at the ends of the lower arms of chromosomes 10 and 20, respectively, is an example of the high rate of recombination between homologous chromatids in chromosome arm end regions.

Our expression analysis showed that the regulation of soybean *Hsp*20 genes is generally associated more with heat stress than with the other tested stresses. A total of 47 Gm*Hsp*20 candidates, including all of the organellar genes, were highly induced under heat shock stress in the roots and leaves, showing variation that ranged from four up to 10,000 times higher expression at 42°C compared with the control condition. The *Hsp*20 chaperone function under heat shock has been elucidated, but the functional roles of these proteins under other stresses or non-stress conditions have not been extensively worked out. The fact that these genes can be induced not only by heat shock but also under other stress conditions, as demonstrated in this study, reflects an interconnected mechanism of induction involving the HSFs. *Hsp*20 genes are known to be specifically controlled by different HSFs, which is interesting considering that there are 52 soybean HSF genes, while other species have closer to 30 HSFs [44,45].

The expression profiles of subfamily CIV and Gm*Hsp*17.7A differed from all of the other clustered nucleocytoplasmic Gm*Hsp*20 genes, mainly because they were not altered by HT, even when the leaves were tested. The tissue-specific expression patterns of *Hsp*20 genes have been reported in different species. In *Arabidopsis*, the expression profile of some At*Hsp*20 genes under heat shock shows great variation depending on the tissue tested [46], while in rice, the expression profiles of the Os*Hsp*18.8-CV and Os*Hsp*19.0-CII genes were shown to be regulated differently in flowers and pistils, respectively [12]. In contrast, our results demonstrate very similar expression profiles of the Gm*Hsp*20 genes among the tissues analyzed under heat shock treatment (four Gm*Hsp*20 and two *Acd* genes).

The Gm*Hsp*22.4, Gm*Hsp*17.9B, Gm*Hsp*17.9A and Gm*Hsp*17.4 genes were induced by *M. javanica* in the susceptible genotype and have been described by Kandoth et al. [6] as also being responsive to *H. glycines* infection (Figure 7). Similarly, four Os*Hsp*20 genes were found to be induced under the biotic stress of infection with *M. grisea* fungus [12]. Similarity analysis revealed that the rice gene *Hsp*16.9A-CI is homologous to Gm*Hsp*17.9B, suggesting that a functional role of this gene, being activated under pathogen infection, might be conserved. Furthermore, two other genes (Gm*Hsp*22.4 and Gm*Hsp*17.6B) are clearly involved in biotic responses. In earlier attempts, Gm*Hsp*17.6B was mapped to a QTL responsible for *Meloidogyne javanica* resistance and displaying a differential expression profile in resistant and susceptible soybean genotypes [3,7]. In our analyses, Gm*Hsp*22.4 was shown to be highly induced in the resistant genotype compared with the susceptible genotype; this gene has been described as being associated with the response to *H. glycines* infection in soybean [12] and as being located near a biotic resistance QTL (http://soybase.org) (Additional file 4: Table S7).

*In silico* analysis of the Gm*Hsp*20 promoter were in line with previous results that reported the occurrence of putative HSEs within −83 bp from the transcription start site in *Hsp*20 genes that are responsive to nematodes. Five Gm*Hsp*20 genes induced by *M. javanica* followed this pattern described by Barcala et al. [14] (Table 1 and Figure 8). Only Gm*Acd*23.1 and Gm*Hsp*19.5, which were induced by nematodes, did not exhibit this pattern.

In *Arabidopsis* mutants for *Hsp*20 genes involved in the responses to nematode infection, the TATAbox element should be preferentially located between 12 and 21 bp upstream of the transcription start site, followed by an HSE at around −83 bp and a CCAAT box between 84 and 141 bp upstream of the transcription start site [14]. The promoter of one *Hsp*20, a CAAT box element was previously reported in the promoter region of Hs1 pro-1, a gene conferring complete resistance to *H. glycines*, and appears to be essential for site-specific regulation [29]. The promoters of all *Hsp*20, which are responsive to nematode infection, also show putative CAAT elements. Moreover, the Gm*Hsp*20 biotic stress-responsive genes followed the same pattern observed in *Arabidopsis* and sunflower and not observed in the others Gmhsp20, where the CAAT box always occurs either between the HSEs or immediately upstream of them, while the W-box, when present, is further upstream of the HSE. However, previous studies have shown the function of these cis-elements in the *Hsp*20 regulation in *Arabidopsis* and sunflower, the involvement of them in soybean responses to nematodes need to be checked by *in vivo* experiments [9,14].

TA-rich elements have been described as being directly involved in the regulation of the expression level of an *Hsp*20 gene in response to nematode infection in soybean [7], and they appear to act by altering the distances between other *cis*-elements, interfering with the strength of the promoter [47]. The number of TA repetitions in the promoter region of a soybean genotype resistant to *M. javanica* appears to be correlated, in a significantly higher level, with Gm*Hsp*17.6B expression observed in response to this stress. The resistant plants contain 32 TA repetitions in the Gm*Hsp*17.6B promoter region, while the susceptible plants have only nine [7]. Our *in silico* analysis showed the occurrence of a putative TA region in the promoter regions of Gm*Hsp*20 responsive to *M. javanica* infection (Gm*Hsp*17.6B, Gm*Hsp*22.4, Gm*Hsp*17.9B and Gm*Acd*23.1). It will be now interesting to investigate if these TA rich regions are really Gm*Hsp*20 cis-elements i.e., if the number of TA repetitions can be correlated to nematode resistance for these genes and if the deletion of TA region can interfere in gene expression.

Two *Acd* genes, Gm*Acd*33.0 and Gm*Acd*23.1, were not induced by heat shock in our analyses, and a sequence comparison showed that these genes exhibit high homology to the rice genes Os*Acd*41.4 and Os*Acd*31.8, respectively. The cellular roles of the *Acd* genes are not very well established, but their homologs in rice and *Arabidopsis* have been shown not to be involved inheat shock responsive (HSR) [12]. These findings, combined with the irregular localization of ACD at the N-terminal ends of the proteins, might suggest that these genes are not real *Hsp*20 genes [48]. Interestingly, however, both genes present a normal HSE distribution in their promoters, and one of them, Gm*Acd*23.1, was induced under biotic stress in the susceptible genotype. Thus, it appears that the *Acd* genes might play roles similar to the constitutive *Hsp*20 genes or could be proteins that are involved in specialized functions.

## Conclusions

This study makes a relevant contribution by identifying 51 potential genes that we suggest compose the soybean *Hsp*20 gene family. The combination of *in silico* prediction strategies and *in vivo* expression analyses showed that the applied bioinformatic tools were very efficient in precisely identifying Gm*Hsp*20 family members. In addition, the Gm*Hsp*20 genes were divided among 13 of the 16 known plant *Hsp*20 subfamilies and two additional unknown subfamilies that showed unique secondary structures and phylogenetic relationships between the soybean subfamily members and with members of the *Hsp*20 gene families identified in other species. We have presented evidence of the genomic complexity and diversity of the expression of the soybean *Hsp*20 gene family. The soybean *Hsp*20 genes are distributed across 17 chromosomes, where gene duplication events have most likely resulted in expansion of the family, most notably for the CI subfamily. The vast majority of the *Hsp*20 genes analyzed *in vivo* (47 genes) were found to be strongly induced under heat shock, but other members of this family could be involved in normal cellular functions, which are unrelated to heat shock. Among the Gm*Hsp*20 genes that were HSR, five were also identified as being involved in the soybean response to cold, and five others were responsive to *Meloidogyne javanica* infection. Furthermore, one predicted Gm*Hsp*20 was shown to be responsive to nematode infection, while no change in expression was observed under other conditions. These genes showed a divergent expression pattern between the examined resistant and susceptible soybean genotypes. The promoter region of the Gm*Hsp*20 members is minimally defined by the presence of a putative TATAbox and one to three putative HSEs, but results obtained elsewhere suggest that other regulatory elements found in this study are also likely to be important, such as W-box, CCAAT box sequences and TA-rich regions. The promoters that were responsive to biotic stress followed the same *in silico* predicted *cis*-element composition and distribution patterns that have been described for other species following nematode infection. Moreover, further investigation is required to obtain clues regarding the functions of the individual genes identified in this study. The results presented here can be further analyzed to reveal candidate genes and promoter structures that will be useful in developing technologies that generate genotypes that are more resistant to the various stresses that affect soybean crops.

## Methods

### Database screening and sequence analyses

The soybean genome annotation database (DB) of Superfamily 1.75 and Phytozome v8.0 (Joint Genome Institute (JGI)) was subjected to Blast searches employing the HMM profile of the *Hsp*20 domain (PF00011) downloaded from PFam (http://pfam.sanger.ac.uk/) to identify *Hsp*20 genes with an e-value ≤ 0.001.

An additional search strategy was to use the word “*Hsp*20” as a keyword to identify gene models annotated as *Hsp*20 in the soybean genome, which could potentially be missed when only the HMM profile is used due to the presence of incomplete domains. Finally, the redundant sequences obtained from both DBs were removed, and a total of 76 candidate soybean *Hsp*20 gene models were returned.

The proteins and 500 bp upstream regions of all predicted genes were searched against the Phytozome database. All predicted proteins were examined for the *Hsp*20 domain using MEME (http://meme.sdsc.edu/meme/cgi-bin/meme.cgi)[49] with the following parameters: repetitions per sequence = 1; maximum number of motifs found = 1; and an ideal motif size between 80 and 100 amino acids [16]. The motif sequence identity was confirmed via analyses using InterProScan (http://www.ebi.ac.uk/Tools/InterProScan/) and PROSITE (http://www.expasy.org).

The protein sequences of the Gm*Hsp*20 candidate genes were evaluated with EXPASY PROTPARAM (http://www.expasy.org/tools/protparam.html) to obtain their molecular weights, theoretical isoelectric points (IP) and instability indices (with a value >40 considered unstable). The chromosomal locations, intron numbers and sizes (bp) were obtained using the Phytozome DB.

The upstream regions (0.5 kb), or 1.5 kb when was necessary, of the *Hsp*20 genes were identified using Phytozome v8.0, and searches for HSEs were performed using the putative *cis-*element databases PlantCare (http://bioinformatics.psb.ugent.be/webtools/plantcare/html/), PLACE (http://www.dna.affrc.go.jp/PLACE/signalup.html), MEME/TOMTOM and MatInspector (Genomatix; http://www.genomatix.de/index.html) [50]. Further analysis to identify conserved motifs, such as CAAT box and W-box sequences, present in the promoter regions of the genes was performed using the same programs.

Digital expression analysis of the *Hsp*20 genes was performed with gene expression evidence search tools against the soybean data available at Genevestigator (https://www.genevestigator.com/gv/plant.jsp) [28], Soybase (http://soybase.org/soyseq/) [51] and the LGE - Soybean Genome Project (http://bioinfo03.ibi.unicamp.br/soja/).

Duplications of *Hsp*20 genes considered parameters 70% identity and 80% coverage. The genes were plotted on chromosomes using MapChart software and physical localization data available at Phytozome. The soybean disease resistance QTLs for nematodes and fungi were retrieved from SoyBase (http://soybase.org, as of Dec. 2011). The physical locations of these QTLs were inferred based on information on the physical locations of markers, which was posted in SoyBase as soybean map version 4.0, and only the QTLs with an associated marker were considered [52].

Specific targeting sequences were predicted with the SignalP program (http://www.cbs.dtu.dk/services/SignalP/), and locations were predicted with Predotar (http://urgi.versailles.inra.fr/predotar/predotar.html) and TargetP, the WoLF PSORT program (http://wolfpsort.org/), MitoProt II - v1.101 (http://ihg.gsf.de/ihg/mitoprot.html) or PTS1 predictor (http://mendel.imp.ac.at/pts1/). The prediction of transmembrane domains was performed with the TMHMM 2.0 program (http://www.cbs.dtu.dk). Multiple sequence alignments were conducted using ClustalX 2.1. A phylogenetic tree was constructed based on ACD amino acid sequences using the neighbor-joining method and bootstrap tests carried out with 1,000 iterations [53]. The obtained trees were viewed using MEGA 5 software. For secondary structure predictions, Phyre^2^ (http://www.sbg.bio.ic.ac.uk/phyre2) was employed.

### Growth conditions and stress treatments

Soybean seeds (*G. max* L, cv BRS 133 and genotype PI 595099, which are susceptible and resistant, respectively, to infection with *Meloidogyne javanica* obtained from the Embrapa Soja Active Germplasm Bank (AGB] were soaked for three days in sand. After stage V3 was reached, the plants were subjected to abiotic or biotic stress in two independent experiments. In the abiotic stress experiments, BR133 genotype plants were exposed to a temperature of 42 ± 2°C (heat stress), or 4 ± 2°C for 3 hours (cold stress), or were maintained at 25 ± 2°C for 3 hours (control plants). After being subjected to these stresses, the leaves were immediately collected in liquid nitrogen and transferred to −80°C. In the biotic stress experiments, the BRS 133 (susceptible) and PI 595099 (resistant) genotypes were inoculated with 500 infective second-stage juvenile (J2) *M. javanica* or not inoculated (control plants). The *M. javanica* eggs were collected as described by Hussey and Barker [54]. The roots were collected at 4 and 8 days post-infection.

### Nematode DNA and plant RNA isolation

Each sample from three repetition blocks, with three replicates, was macerated separately using a pestle, mortar and liquid nitrogen. After maceration, the samples were distributed into 1.5 mL microtubes and stored at −80°C.

After the experiment, to obtain molecular verification of nematode infection, we performed DNA extraction and subjected the DNA to PCR, according a protocol described by Rahmanet al. [55] (Additional file 1: Figure S9). A specific oligonucleotide primer set was used to detect *M. javanica* infection, resulting in an amplicon of 945 bp (*foward*_5′-CAAAACCACGCGGCTTCGGC-3′ and *reverse*_5′-TGGGGGTGCCCTTCCGTCAA-3′).

Total RNA (1 μg) was isolated from frozen roots, that were infected with *M. javanica* or not infected, and from samples subjected to abiotic stress treatment using an RNA extraction kit with the TRIzol® reagent (*Invitrogen*, Carlsbad, CA, USA). Quantification and quality analysis were performed with an Uniscience NanoDrop ND-1000 spectrophotometer (NanoDrop Technologies, Wilmington, DE, USA) at a wavelength of 230 nm or via agarose gel electrophoreses, respectively, and the RNA samples were treated with deoxyribonuclease I (Kit DNaseI, Invitrogen) (Additional file 1: Figure S10).

To synthesize cDNA from treated RNA, we used the SuperScript™ III Kit (Invitrogen) according to the manufacturer’s instructions and stored the cDNA at −20°C. Validation of the quality of the cDNA samples was performed using PCR with primers designed to anneal to two different exons of the β-actin gene (*forward*_ 5′-CCCCTCAACCCAAAGGTCAACAG-3′ and *reverse*_ 5′-GGAATCTCTCTGCCCCAATTGTG-3′) (Additional file 1: Figure S11).

### qRT-PCR

The expression profiles of the Gm*Hsp*20 gene models and the *Acd* genes were evaluated under abiotic and biotic stress conditions using qRT-PCR. Primers specific to each of the 51 candidate gene models and two *Acd* genes were designed using the software Primer3Plus (http://www.bioinformatics.nl/cgi-bin/primer3plus/primer3plus.cgi) and Vector NTI Advance™ (*Invitrogen*). The sequences of the primers are listed in Additional file 5: Table S8.

The cDNA samples were amplified with primers specific to each gene model and for the β-actin gene as endogenous control, at a final concentration of 0.1-0.25 μM, with the 1X SYBR Green Master Mix Kit (Applied Biosystems) in a final volume of 12.5 μL. The E = [10^-1^/slope]^-1^ formula was employed to calculate the reaction efficiency and to adjust the final primer concentration. The calibration curve was established based on the Ct and the log of the cDNA dilutions. The reactions were performed in a 7300 qRT-PCR thermocycler (Applied Biosystems) following the manufacturer’s instructions. After initial steps at 50°C for 2 min (UNG activity) and at 95°C for 10 min (activation of the Ampli Taq Gold polymerase), a two-step program of 95°C for 15 s and 62°C for 1 min was run for 40 cycles. Dissociation curves were obtained to guarantee the absence of nonspecific amplification. The data were collected in the log phase, and the results were analyzed with the Sequence Detection program (Perkin Elmer, Waltham, Massachusetts, U.S). The final relative quantification of each gene compared with the control conditions was estimated considering the RQ obtained in each biological replicate, represented by each independent experiment, with three replicates each. Significant differences were determined based on estimates of the standard deviation (SD) and with REST software version 2.0.7 (p < 0.05) (http://gene-quantification.eu/chapter-3-pfaffl.pdf).

### Screening for putative TFBS (transcription factor binding site) combinatorial models

The results of the *in silico* and *in vivo* expression profile analyses of the Gm*Hsp*20 candidates under biotic and abiotic stress conditions were used to determine TFBS combinatorial models for their promoters. These searches were conducted using the FrameWorker – Genomatix suite of programs (http://www.genomatix.com; Germany). The 500 bp region upstream of the promoter region was analyzed for each gene.

## Abbreviations

ACD: Alpha crystallin domain; dpi: Days post-inoculation; GmHsp20: *Glycine max* heat shock protein 20; HMM: Hidden Markov model; HSE: Heat shock element; HSPs: Heat shock proteins; Hsp20: Heat shock protein 20 (or small heat shock proteins); HSR: Heat shock responsive; HT: High temperature; HS: Heat shock; HSF: Heat shock factor; MYB: Myeloblastosis Oncogene; NPR: Non-expressor of pathogenesis related; qRT-PCR: Quantitative real-time polymerase chain reaction; QTL: Quantitative trait locus; TF: Transcription factor; TFBS: Transcription factor binding site.

## Competing interests

The authors declare that they have no competing interests.

## Authors’ contributions

VSLC, MCCGC and FCMG planned and designed the study. VSLC performed the computational analysis, executed the experiments, generated the figures and drafted the manuscript. MCCGC, LMD and MKK also contributed to the execution of the abiotic and biotic experiments. LMD and MKK also contributed to samples preparation. WPD provided the nematode material and contributed to leading the biotic experiment. VSLC, MCCGC and FCMG performed the qRT-PCR data analysis. MCCGC, FCMG, ALN and RVA contributed to the Discussion of the manuscript. All authors read and approved the final manuscript.

## Supplementary Material

Additional file 1: Figures S1-S11 Results for ACD domain (S1); Heatmap for microarray from Genevestigator (S2); Logos for HMM to HSEs (S3), HSE sites by MatIsnpector (S4); HSE sites by PLACE (S5); Electrophoresis for primers test PCRs (S6); Electrophoresis for expression induction evidence by conventional PCR (S7), Venn Diagram for common and exclusive expressed genes (S8); Electrophoresis for nematode infection evidence (S9); Electrophoresis for RNA extracted integrity (S10); Electrophoresis for cDNA samples quality analysis (S11). Click here for file

Additional file 2: Tables S1-S5 Summary of the Gm*Hsp*20 genes in soybean (S1); ACD and HSE predicted position (S2); Subcellular localization (S3); Predicted Physicochemical features (S4); RTq-PCR DATA summary (S5). Click here for file

Additional file 3: Table S6 Soybean *Hsp*20 Gene Family Duplication Analysis. Click here for file

Additional file 4: Table S7 Resistance QTL in the 2 Mb flanking region of Gm*Hsp*20. Click here for file

Additional file 5: Table S8 Information on the qRT-PCR primers used for expression analysis. Click here for file
