# Supplementary material for: Genome-wide analysis of the Hsp20 gene family in soybean: comprehensive sequence, genomic organization and expression profile analysis under abiotic and biotic stresses
Source: BMC Genomics. 2013 Aug 28;14:577. doi: 10.1186/1471-2164-14-577 (PMC3852298; doi:10.1186/1471-2164-14-577)
Supplement: Additional file 1: Figures S1-S11 — Results for ACD domain (S1); Heatmap for microarray from Genevestigator (S2); Logos for HMM to HSEs (S3), HSE sites by MatIsnpector (S4); HSE sites by PLACE (S5); Electrophoresis for primers test PCRs (S6); Electrophoresis for expression induction evidence by conventional PCR (S7), Venn Diagram for common and exclusive expressed genes (S8); Electrophoresis for nematode infection evidence (S9); Electrophoresis for RNA extracted integrity (S10); Electrophoresis for cDNA samples quality analysis (S11). [file 1471-2164-14-577-S1.docx]

**Additional file 1_Suplemental Figures S1 to S12**

**
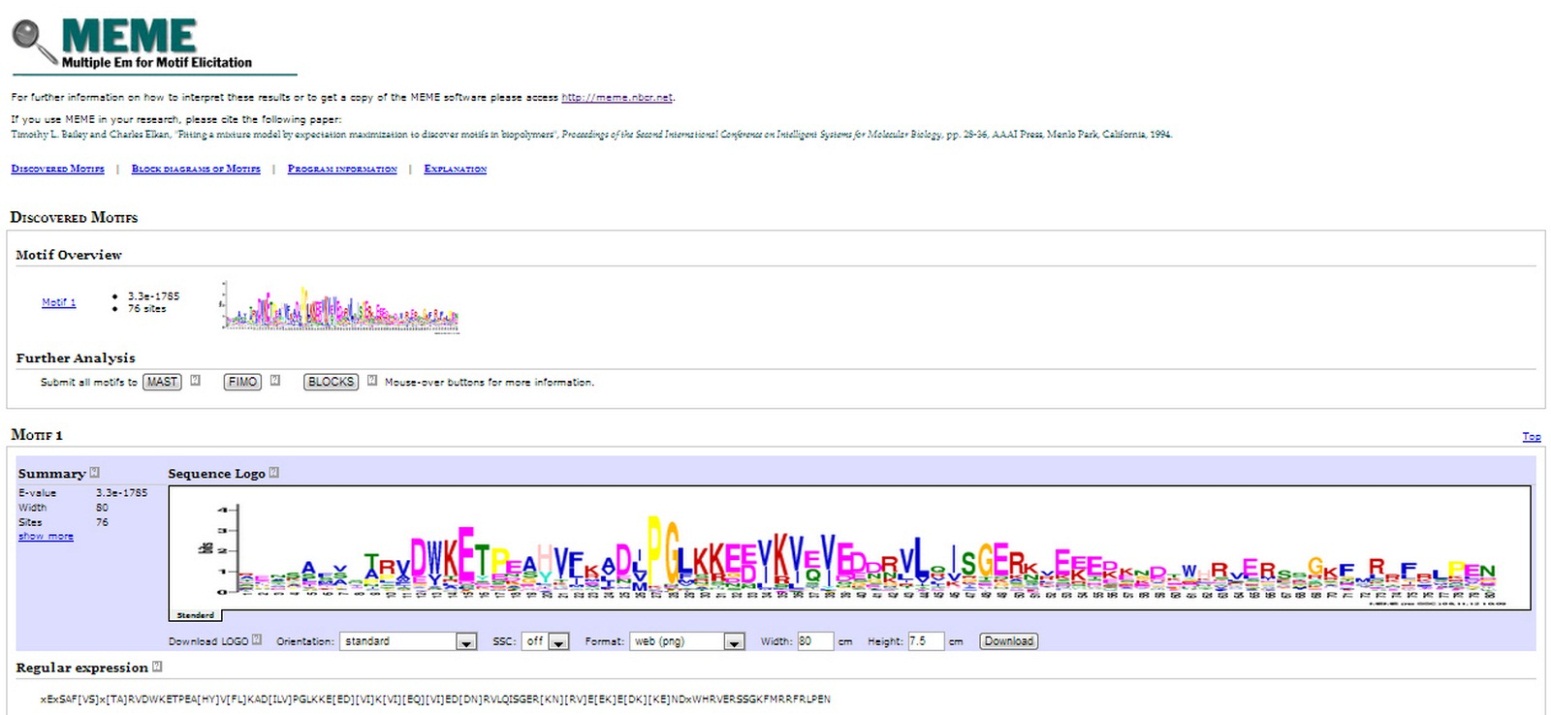
**

**Figure S1: Logo obtained in MEME/MAST for HMMto ACD domain (3.3e^-1785^) found in putative GmHsp20 proteins**. The motif e-value is an estimate of the expected motifs number with the same size and occurrences that would be present in a set of random sequences similar size. The symbols height in each motif amino acid position indicates the conservation sequence.It were obtained a graphic map by MEME analysissearchingfor ACD on each putative GmHsp20 proteins. The blue bars indicate the ACD field position in each 76 GmHsp20 candidates. In the scale, underside at figure part, the number 0 indicates the N-terminal region and the 400 C-terminal region. The p-values ​​are found just earned column (lowest p-valeu) on right column glymas. (name).


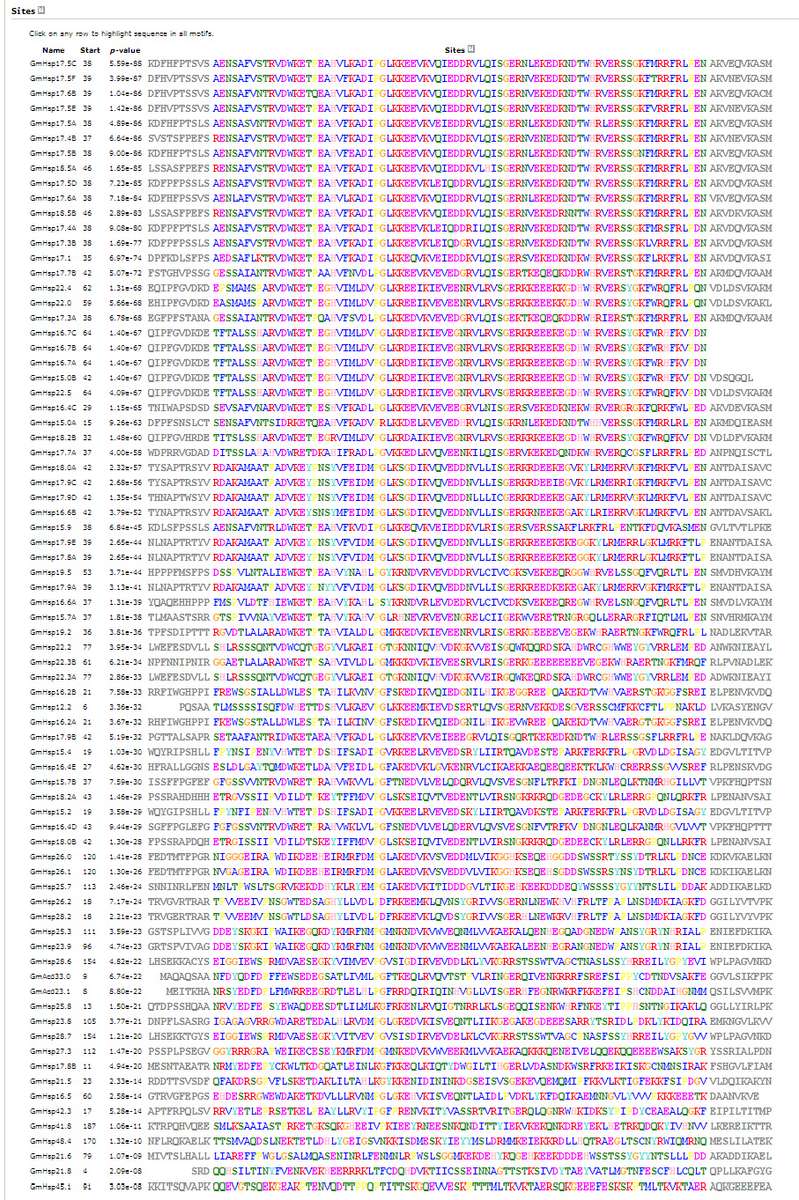
**Figure S1: Complement.**


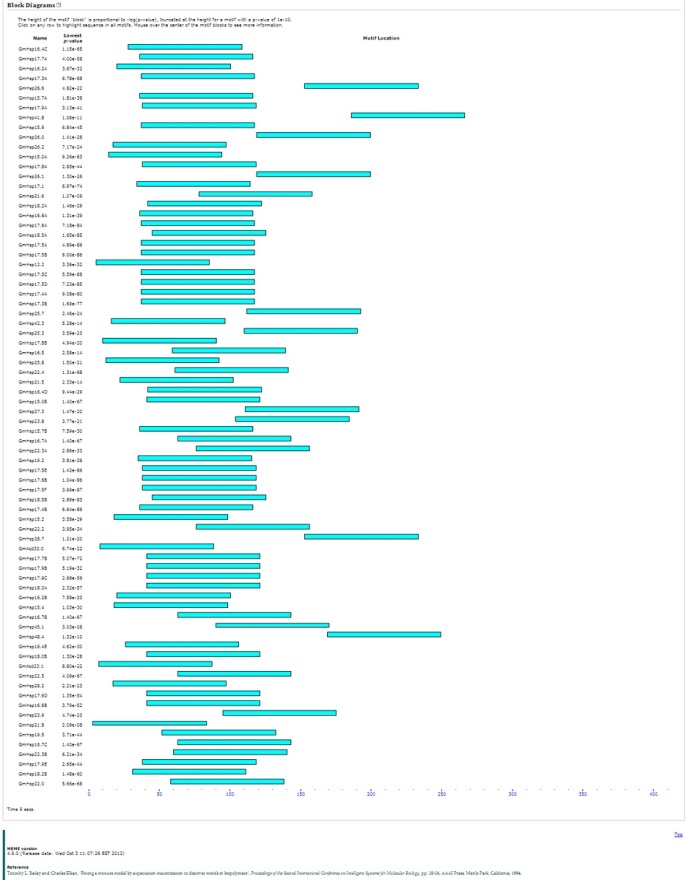
**Figure S1: Complement.**

**
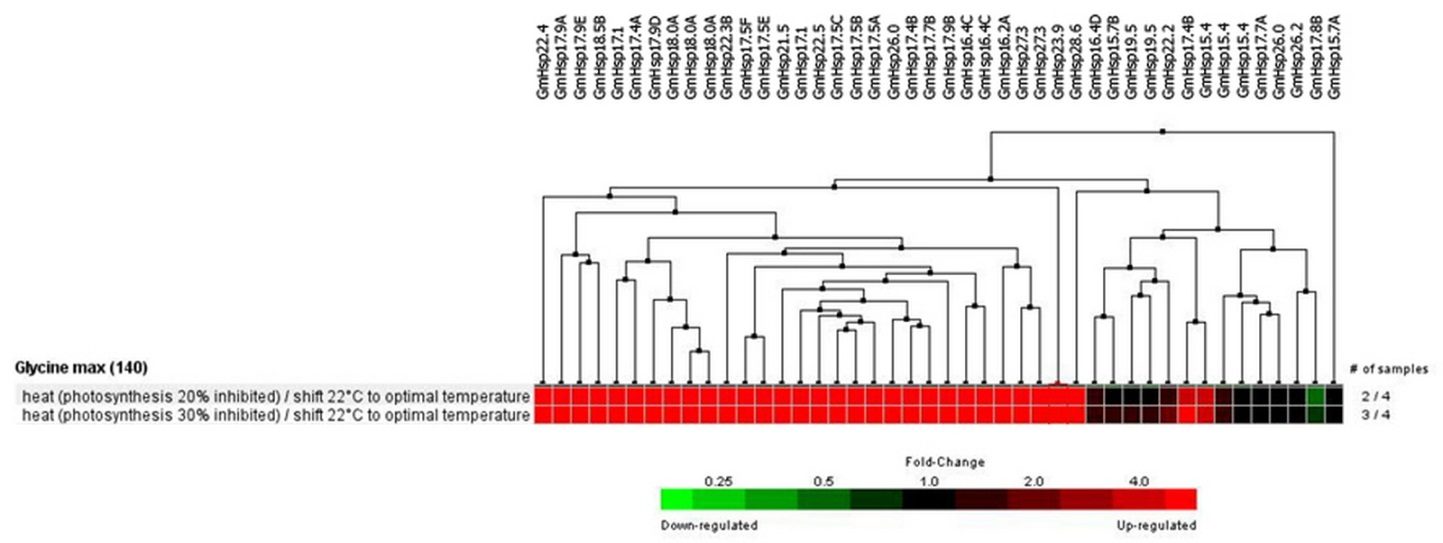
Figure S2: Heatmap for microarray experiments based on GmHsp20 genes expression profiles during HT, from Genevestigator database.** The Hsp20 gene expression during heat stress are presented as heat maps in red/black/green gradient using meta-analysis tool http://www.genevestigator.ethz.chGenevestigator. The darker red color corresponds to up-regulated expression, while theblack color corresponds to no significant expression difference between treatments and thegreen color corresponds to down-regulated expression. The existence of more than one probe for a gene explains the presence in some instances of the same gene model be repeated two or more times.


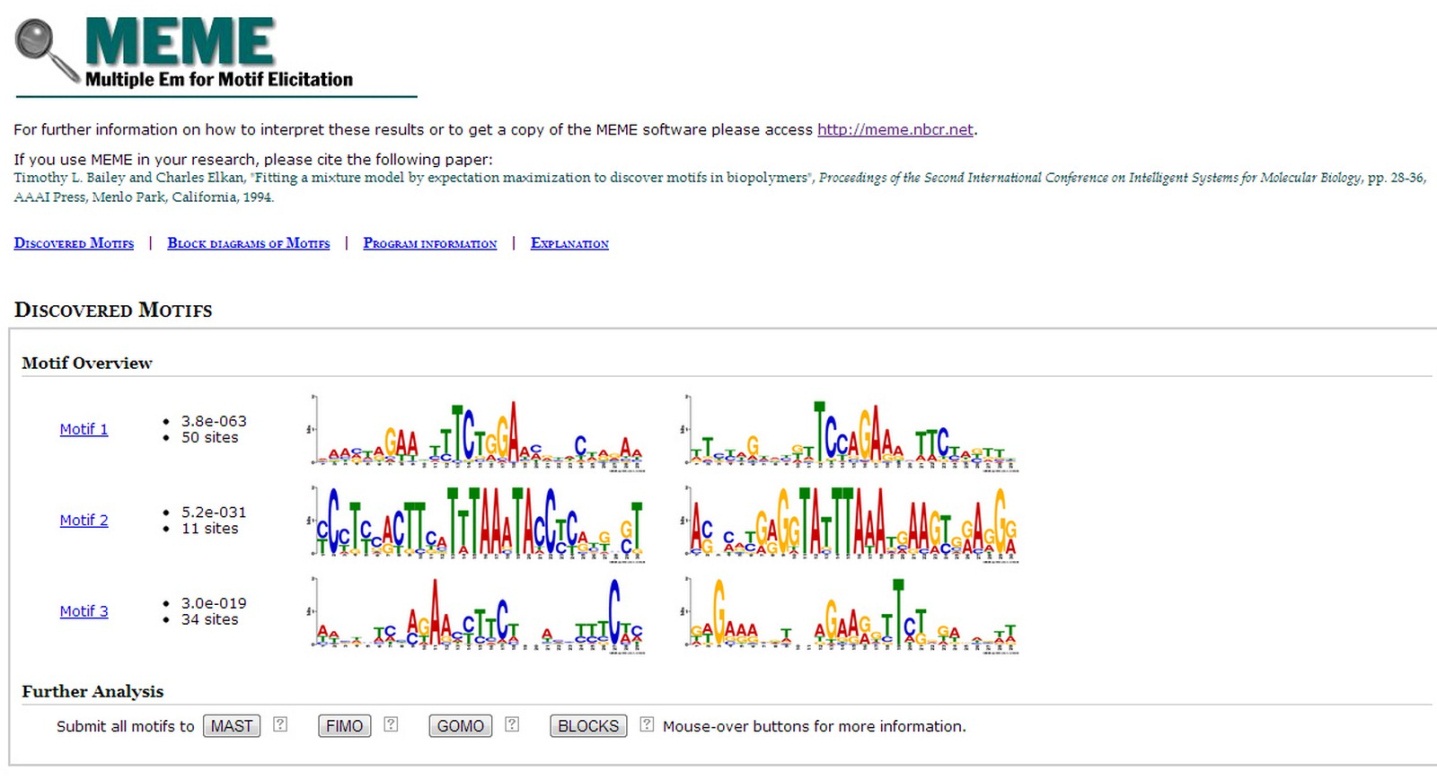
**Figure S3: Logos obtained in MEME/TOMTOM for HMM to HSEs (3.3e^-1785^) found in putative GmHsp20 upstream sequence**. The motifs e-value is an estimate of the expected motifs number with the same size and occurrences that would be present in a set of random sequences similar size. The symbols height in each motif nucleotide position indicates the conservation sequence. The motifs 1 and 3 were identified as HSE and showed an e-valeu 3.3 e^-063^ and 3.o e^-019^respectively. The colored bars indicate the HSEs field position in each GmHsp20 candidates with upstream sequence predicted at Phytozome. In the scale, underside at figure part, the number 0 indicates the 500bp upstream at transcriptional start site and the 500 indicates the 1bp upstream. The p-values ​​are found just earned column (lowest p-valeu) on right column glymas. (name). Only the motifs found on the tape were considered positive, following the guidance of reading the gene.


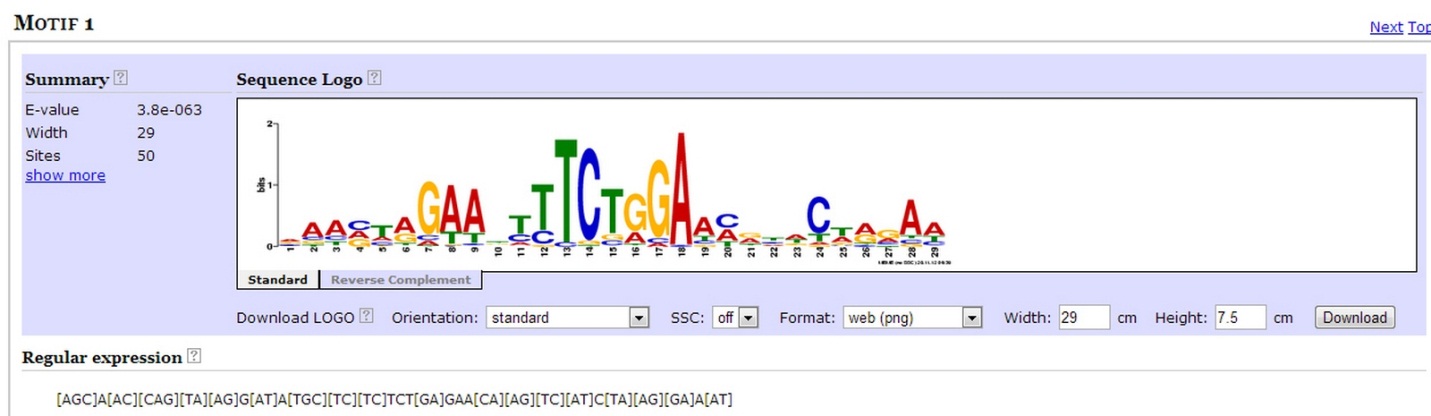
**Figure S3: Complement.**

**
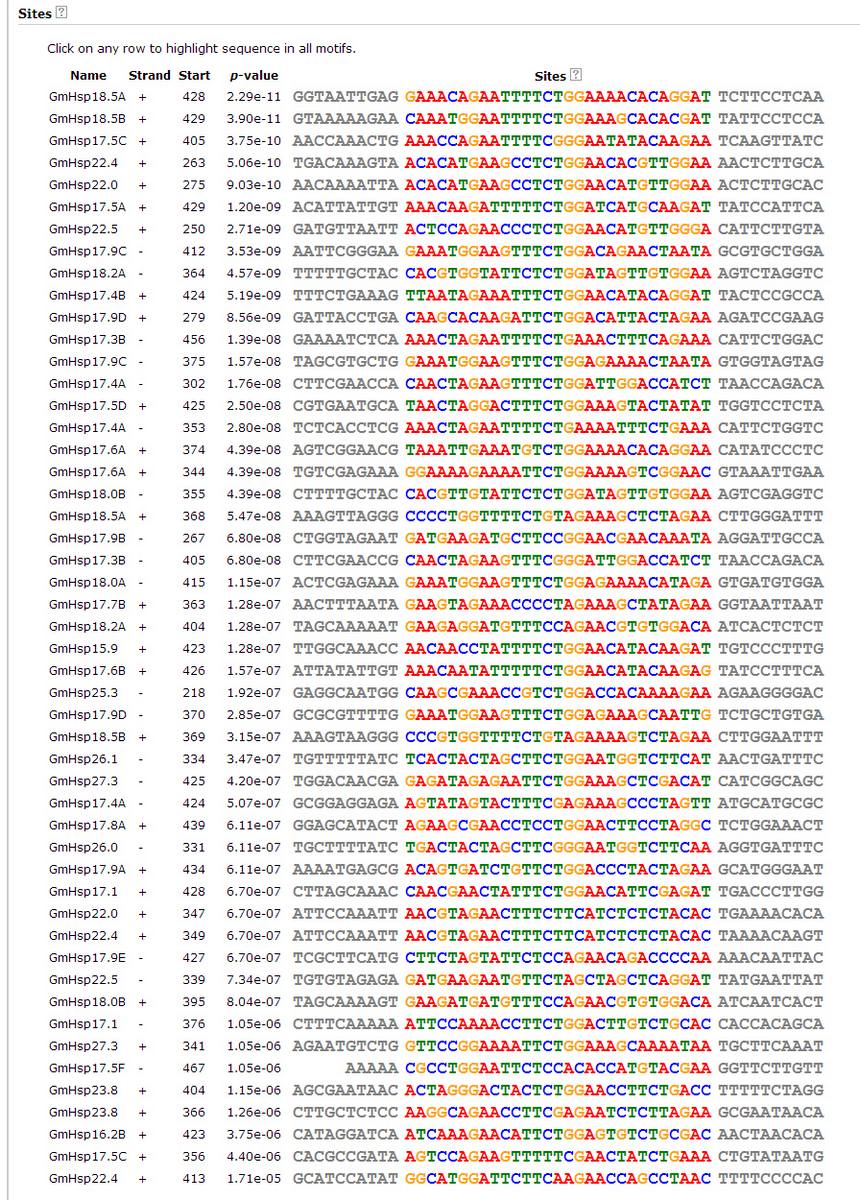
Figure S3: Complement.**

**Figure S3: Complement.
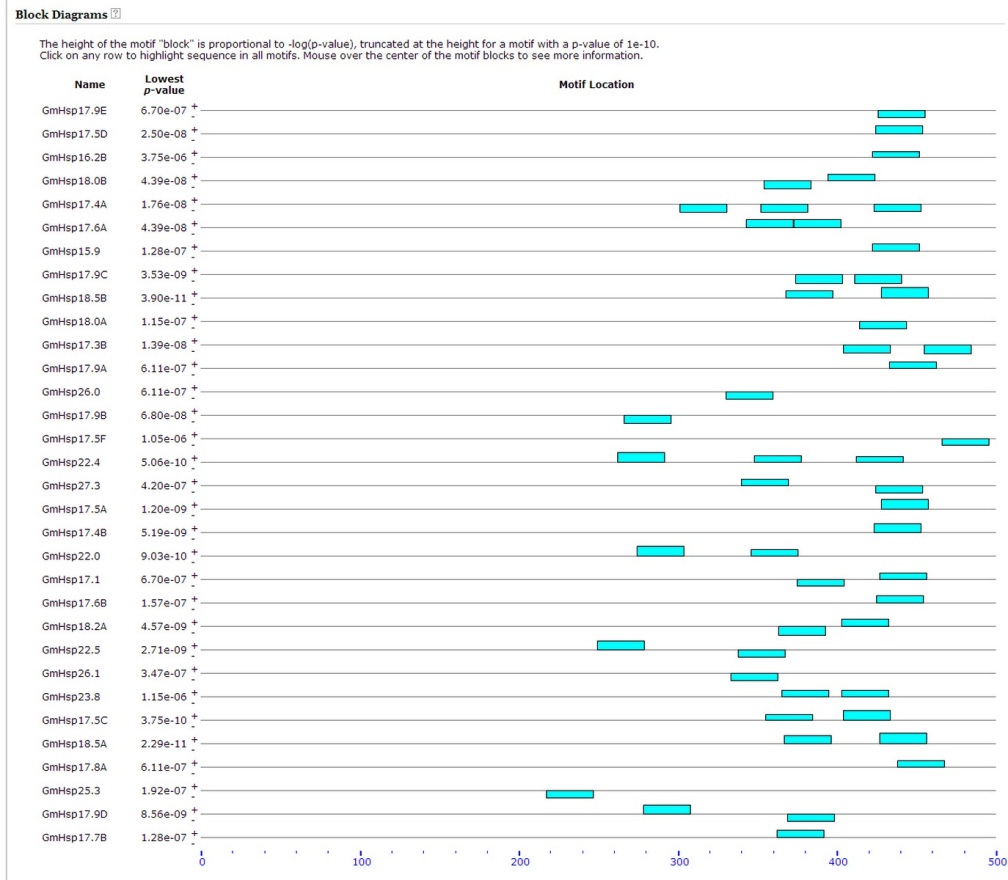
**

**
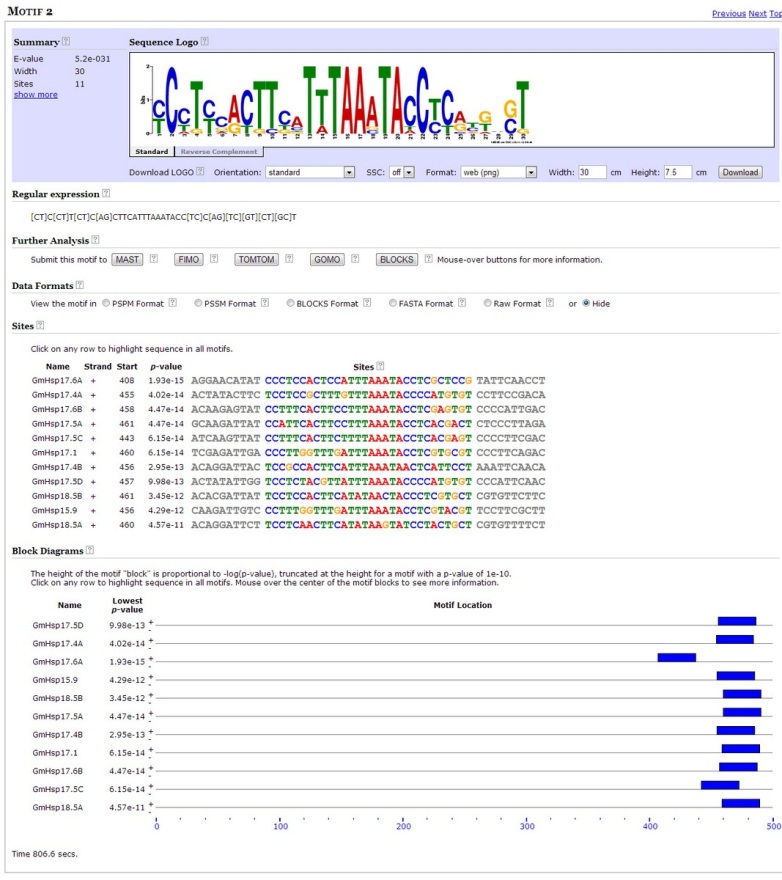
Figure S3: Complement.**

**
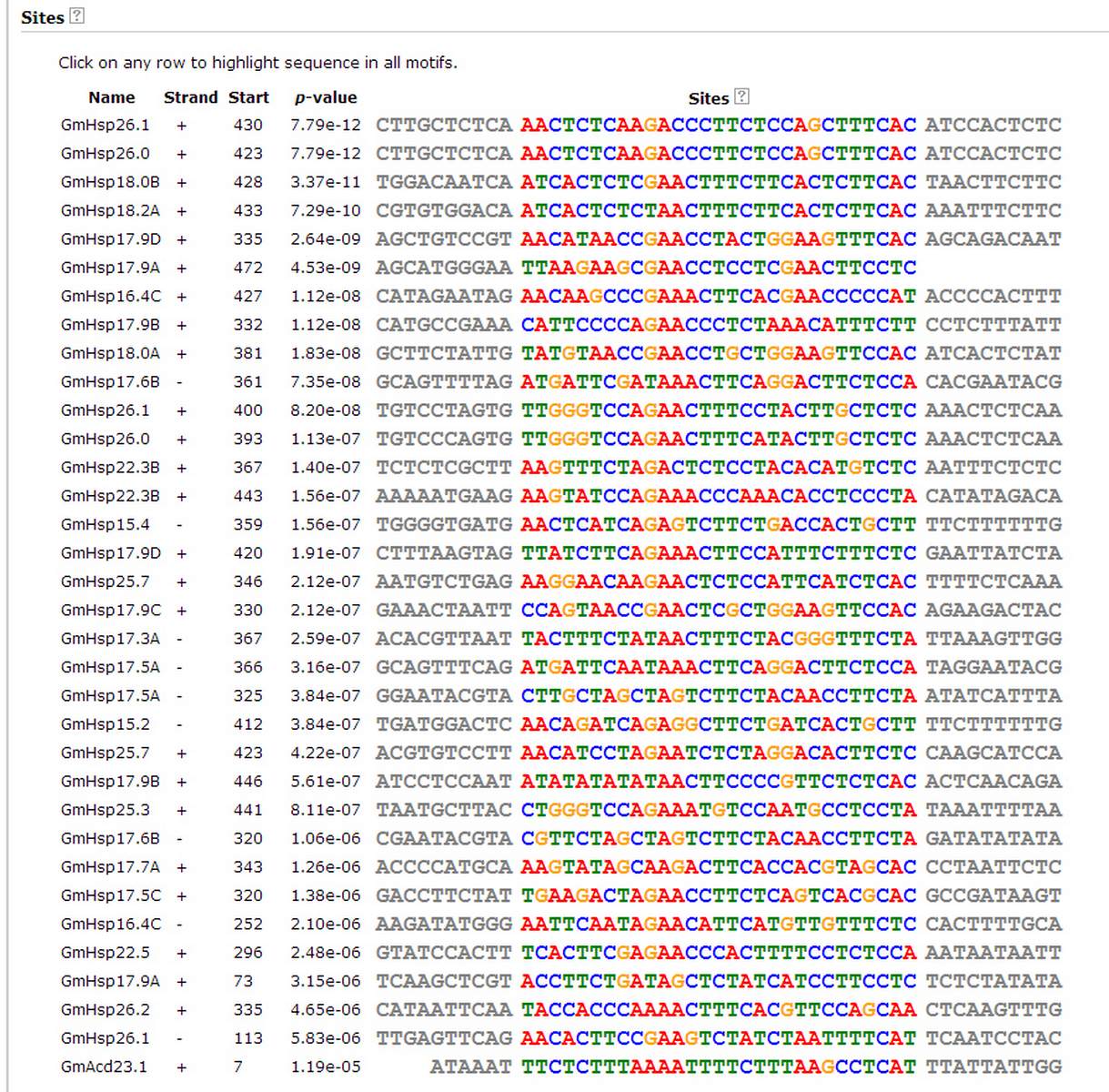
Figure S3: Complement.**

**Figure S3: Complement
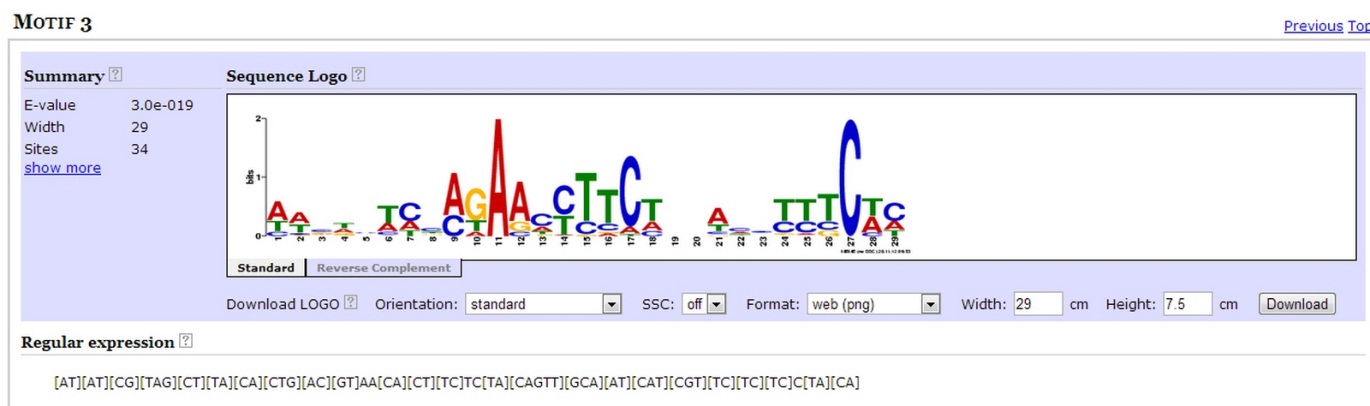
.**

**Figure S3: Complement.**

**
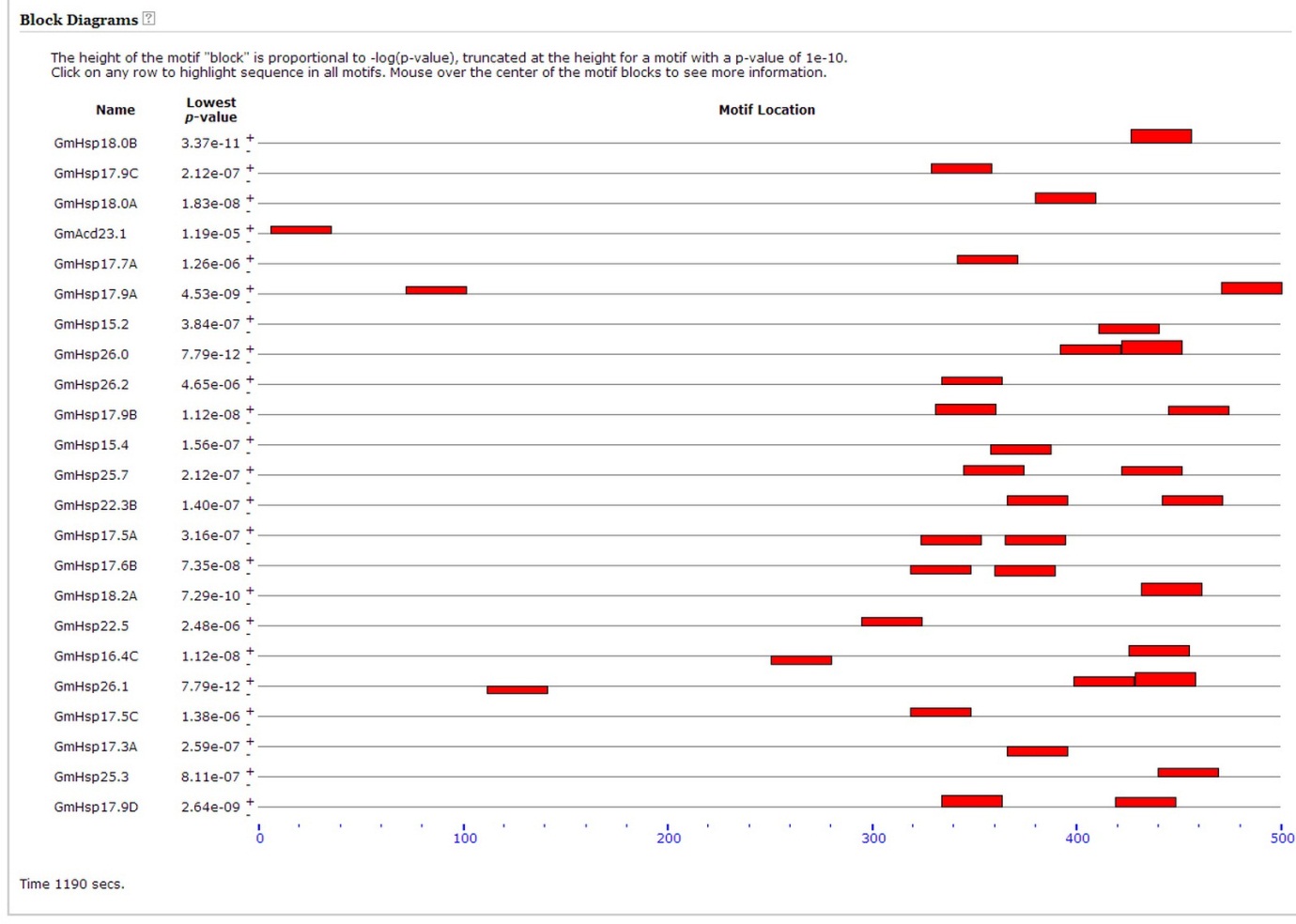
**

**
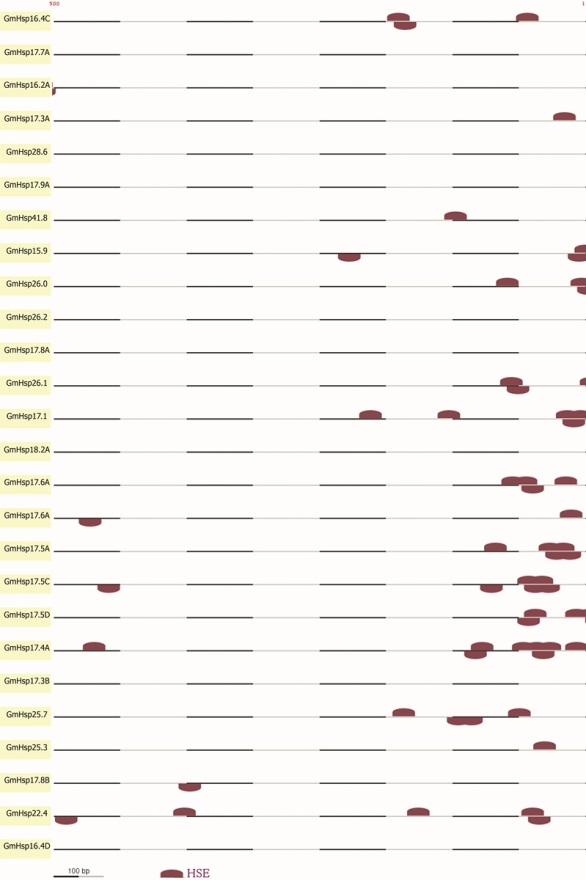
Figure S4: Illustration forheat shock transcription factor binding sites predicted by MatIsnpector software.** It were analyzed thepromoter regions of each GmHsp20 candidates with upstream sequence predicted at Phytozome. For some promoters, the putative HSEs were found at outside positions the standards usually found to Hsp20. Were analyzed up to 500pb, or 1500 bp, upstream to transcription start site predicted. The brown marks on the tapes during indicate the position of HSE in each sequence. In the scale number 1 indicates -1 bp of the transcription start site and the number 500/1500indicates 500bp/1500bp upstream to transcription start site predicted. Only the motifs found on the tape were considered positive, following the guidance of reading the gene.

**
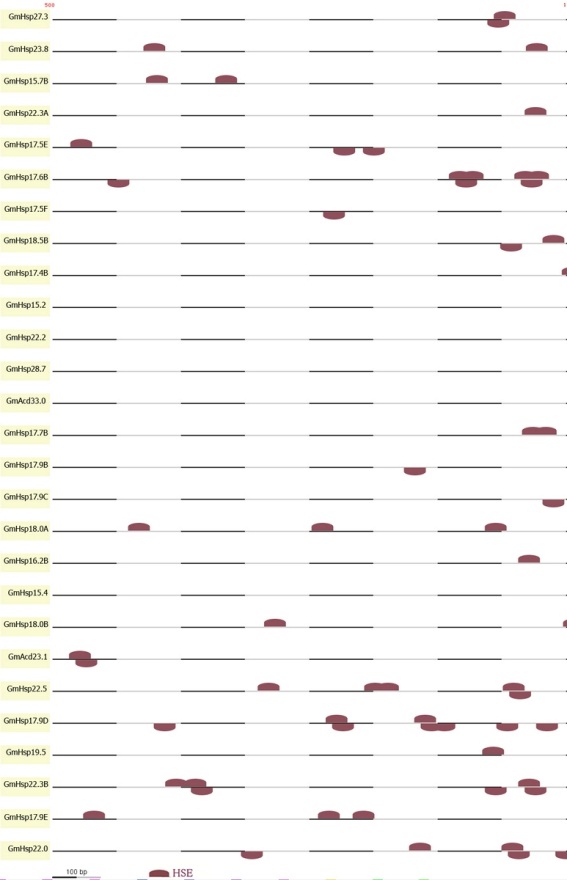
**

**Figure S4: Complement.**

**Figure S4: Complement.**

**
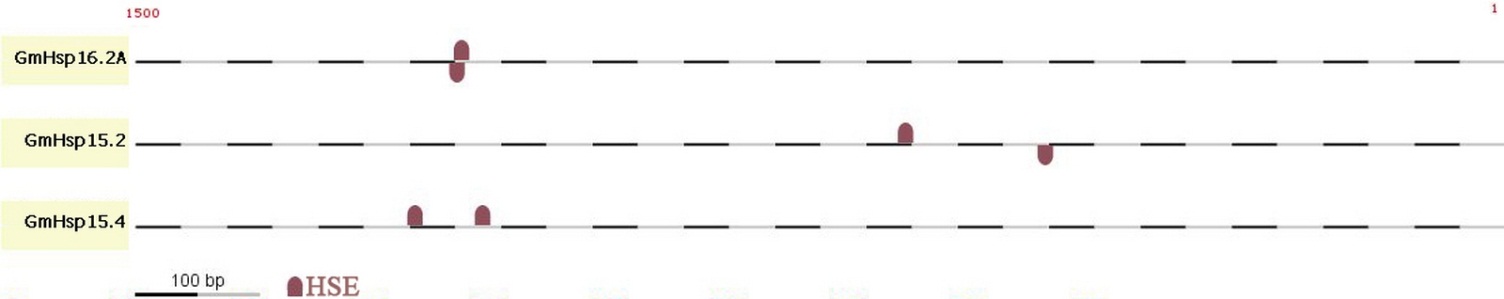
**

**
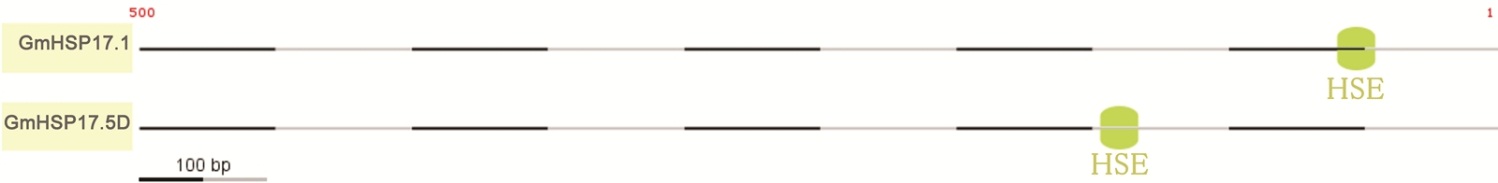
Figure S5: Illustration for heat shock transcription factor binding sites predicted by PLACE tool.** It were analyzed the promoter regions of each GmHsp20 candidates with upstream sequence predicted at Phytozome. For some promoters, the HSEs were found at outside positions the standards usually found to Hsp20. Were analyzed up to 500pb, or 1500 bp, upstream to transcription start site predicted. The green marks on the tapes during indicate the position of HSE in each sequence. In the scale number 1 indicates -1 bp of the transcription start site and the number 500/1500 indicates 500bp/1500bp upstream to transcription start site predicted. Only the motifs found on the tape were considered positive, following the guidance of reading the gene.


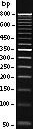


b


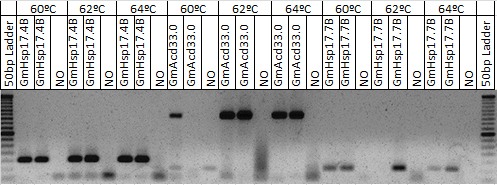

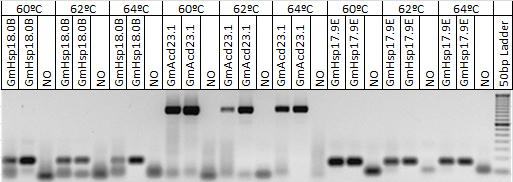


a

**Figura S6: Primers test using soybean DNA as template.**Electrophoresis on 1.2% agarose gel of the PCR reaction products to amplification assays (pairing specificity and temperature) of the primers to genomic DNA from soybean. a.The results of oligonucleotides testing for gene models GmHsp17.9E, GmHsp17.4B, GmAcd33.0, GmAcd23.1, GmHsp18.0B and GmHsp17.7B. The first header lines of each picture are annotated tested temperatures for each primer pair, and the second line followed by primers tested negative reaction controls (NO). We used molecular marker of 50bp (50bp Ladder - Invitrogen). b. Ladder 50bp - Invitrogen. Horizontally, are indicated sizes in base pairs, the major bands of the marker.

**
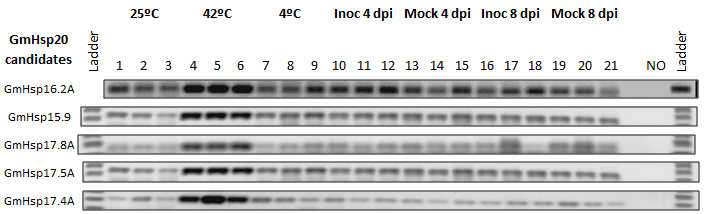
**

🡸100bp

🡸100bp

🡸100bp

🡸150bp

🡸150bp

**Figure S7**: **Expression induction evidence by conventional PCR under Heat Shock**. Electrophoresis on 1.2% agarose gel of conventional PCR products targeting the cDNA.This figure are the results six specific primers for GmHsp20 candidates: GmHsp16.2A, GmHsp15.9, GmHsp17.8A, GmHsp18.2A, GmHsp17.5A and GmHsp17.4A, as an example of Hsp20 genes induced by heat induced observed by conventional PCR analyses. The first photo header, shows the gene analyzed. The first photo header, shows each treatment, 25°C (control treatment tempetratura), 42°C (heat shock treatment), 4 (cold treatment), Inoc 4dpi (M.javanica treatment 4 days after inoculation), 4dpi Mock (M.javanica treatment 4 days after falseinoculation), Inoc 8dpi (M.javanica treatment8 days after inoculation), 8 dpi Mock (M.javanica treatment8 days after false inoculation), all in three biological repletions, and negative control reaction (NO). We used a molecular marker of 100 base pairs (Ladder 100 bp - Invitrogen).

**
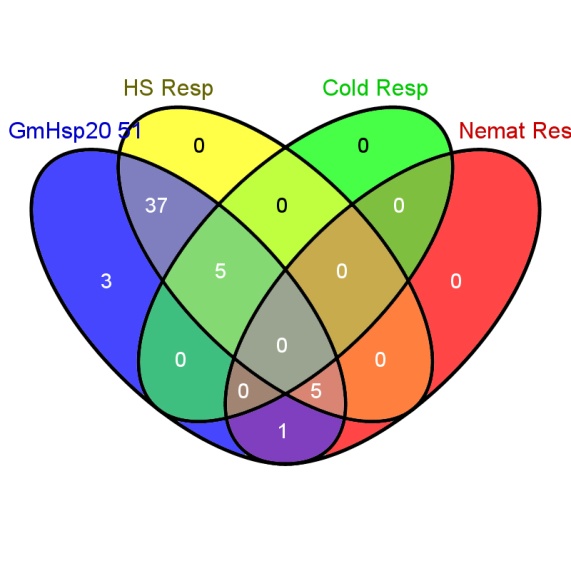
**

**Figure S8: Venn Diagram to expression profile distribution of GmHsp20 candidates in treatments.** GmHsp20 51: consist in all of the candidates; HS Resp: all of the candidates that were heat stress responsive; Cold Resp: all of the candidates that were coldstress responsive; NematResp: all of the candidates that were biotic (*M. javanica*) stress responsive.

**
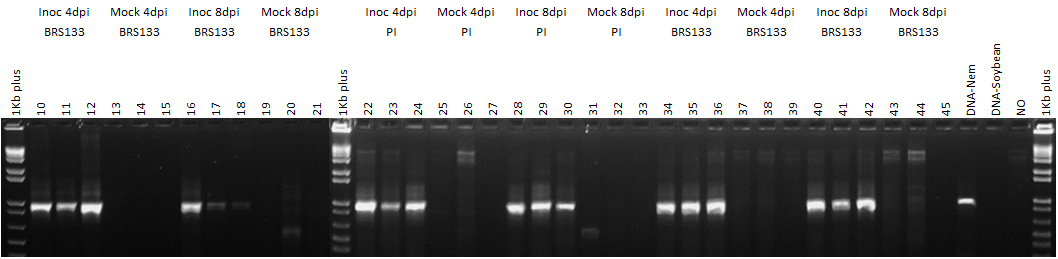
Figure S9: Nematode infection evidence.** Electrophoresis on 1.2% agarose gel of PCR products, with 945bp, to confirm the soybean plants infection by *M. javanica*. The first header line indicate the samples and the negative control reaction (NO). We used the 1kb plus molecular marker (1 kb plus ladder - Life Technologies). b. Ladder 1 kb plus - life technologies. Horizontal are indicated some markers size, in base pairs.


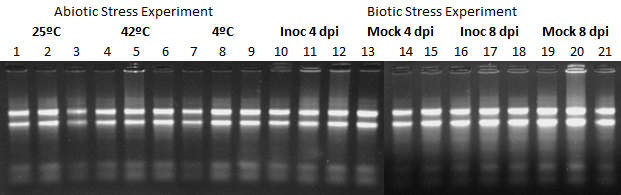


**Figure S10: RNA extracted integrity result**. Electrophoresis on 1% agarose gel to analyze the total RNA integrity samples extracted from biotic and abiotic stress treatments. The first photo (top) shows the BRS133 (1 to 9) RNA samples from abiotic stress, and BRS133 (10 to 21) RNA samples from biotic stress (infection by *M. javanica*). The second photo (below) shows just biotic stress (infection by *M. javanica*) RNA samples. The PI595099 (22 to 33) RNA samplesand BRS133 (34 to 45) RNA samples, both from biotic stress.


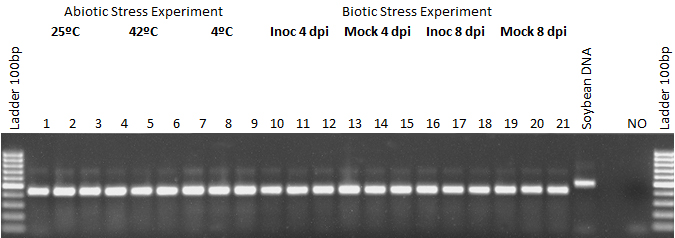


**
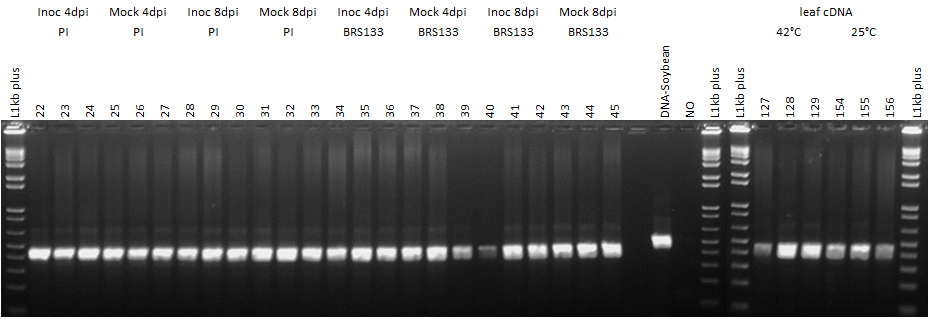
Figure S11: cDNA samples quality results.** Electrophoresis on 1.2% agarose gel of PCR products using primers set for β-actin gene with synthesized cDNA samples as template. The expected amplicon size has 520 bp to genomic DNA as template and 440 bp when RNA as template. The second photo (below) shows just biotic stress (infection by *M. javanica*) cDNA samples. The PI595099 (22 to 33) cDNA samples and BRS133 (34 to 45).
